# A Keratin 7 and E-Cadherin Signature Is Highly Predictive of Tubo-Ovarian High-Grade Serous Carcinoma Prognosis

**DOI:** 10.3390/ijms22105325

**Published:** 2021-05-18

**Authors:** Laudine Communal, Noemi Roy, Maxime Cahuzac, Kurosh Rahimi, Martin Köbel, Diane M. Provencher, Anne-Marie Mes-Masson

**Affiliations:** 1 Institut du Cancer de Montréal, Montreal, QC H2X 0A9, Canada; laudine.communal@gmail.com (L.C.); noemi.roy@hotmail.com (N.R.); maxime.cahuzac@umontreal.ca (M.C.); kurosh.rahimi.chum@ssss.gouv.qc.ca (K.R.); diane.provencher.chum@ssss.gouv.qc.ca (D.M.P.); 2 Centre de Recherche du Centre Hospitalier de l’Université de Montréal (CRCHUM), Montreal, QC H2X 0A9, Canada; 3 Department of Pathology, Centre Hospitalier de l’Université de Montréal (CHUM), Montreal, QC H3T 1J4, Canada; 4 Department of Pathology and Laboratory Medicine, University of Calgary, Calgary, AB T2N 1N4, Canada; martin.kobel@cls.ab.ca; 5 Division of Gynecologic Oncology, Université de Montréal, Montreal, QC H3T 1J4, Canada; 6 Department of Medicine, Université de Montréal, Montreal, QC H3T 1J4, Canada

**Keywords:** epithelial tubo-ovarian high-grade serous carcinoma, Keratin 7/Cytokeratin 7/KRT7/CK7, E-Cadherin/Cadherin 1/E-CADH/CDH1, prognosis biomarker, predictive biomarker, epithelial to mesenchymal transition

## Abstract

During tubo-ovarian high-grade serous carcinoma (HGSC) progression, tumoral cells undergo phenotypic changes in their epithelial marker profiles, which are essential for dissemination processes. Here, we set out to determine whether standard epithelial markers can predict HGSC patient prognosis. Levels of E-CADH, KRT7, KRT18, KRT19 were quantified in 18 HGSC cell lines by Western blot and in a Discovery cohort tissue microarray (TMA) (*n* = 101 patients) using immunofluorescence. E-CADH and KRT7 levels were subsequently analyzed in the TMA of the Canadian Ovarian Experimental Unified Resource cohort (COEUR, *n* = 1158 patients) and in public datasets. Epithelial marker expression was highly variable in HGSC cell lines and tissues. In the Discovery cohort, high levels of KRT7 and KRT19 were associated with an unfavorable prognosis, whereas high E-CADH expression indicated a better outcome. Expression of KRT7 and E-CADH gave a robust combination to predict overall survival (OS, *p* = 0.004) and progression free survival (PFS, *p* = 5.5 × 10^−4^) by Kaplan–Meier analysis. In the COEUR cohort, the E-CADH-KRT7 signature was a strong independent prognostic biomarker (OS, HR = 1.6, *p* = 2.9 × 10^−4^; PFS, HR = 1.3, *p* = 0.008) and predicted a poor patient response to chemotherapy (*p* = 1.3 × 10^−4^). Our results identify a combination of two epithelial markers as highly significant indicators of HGSC patient prognosis and treatment response.

## 1. Introduction

Tubo-ovarian high-grade serous carcinoma (HGSC) is the most frequent, aggressive, and lethal histotype among ovarian carcinomas (OC). HGSC originates most frequently from the fimbrial mucosa of the Fallopian tubes and rapidly disseminates throughout the peritoneal cavity [1,2]. HGSC standard management consists of cytoreductive surgery and platinum and taxol-based chemotherapy but most patients will relapse within five years [3]. Molecular subtypes of HGSC have been identified in recent years, but their association with patient prognosis and response to therapy remains uncertain [4,5,6,7,8]. Therefore, there may be a greater value in finding consensus biomarkers that do not belong in a specific molecular subtype but that are able to stratify patients by prognosis and likely treatment responsiveness in order to tailor new therapeutic strategies for patients who are unlikely to respond to standard care [8]. Tumor profiling and biomarker signatures also provide indications of tumor phenotypes and activated signaling pathways that could be targeted by specific and personalized treatments [4,9,10].

Epithelial cell plasticity has been extensively described in the literature with respect to the capacity of tumor cells to navigate between an epithelial to mesenchymal phenotype or to regain stemness potency [9]. Epithelial phenotype is characterized by a plethora of markers, depending on the observed tissue and the type of epithelium. E-cadherin (E-CADH) and keratins (KRTs), such as the type II keratin 7 (KRT7) and the type I keratins 18 (KRT18) and 19 (KRT19), are commonly used to characterize the tumoral cells in HGSC tumors [10,11]. E-CADH has been frequently found downregulated in malignant epithelial tumors compared to their normal tissue counterparts, and high levels of E-CADH protein have been associated with a favourable prognosis in HGSC [11,12,13,14]. KRTs are known constituents of the cytoskeleton intermediate filaments in epithelial cells and are routinely used by pathologists as epithelial tumors that largely maintain the keratin profiles associated with their respective cells of origin [15,16,17]. KRT7, KRT18, KRT19 protein expression in tumors were reported to predict prognosis in several cancer types [18,19,20,21,22]. However, despite reported functions of KRTs in tumor progression, relatively little is known about their value as prognostic markers in the context of HGSC [17,23,24,25]. Interestingly, we previously showed that KRT7 and KRT19 mRNA were elevated in OC compared to borderline tumors [26]. The relative levels of KRTs and E-CADH in HGSC may have an impact on tumor biology and patient prognosis.

In the present study, we focus on epithelial marker variability in HGSC and whether it is associated with aggressiveness of the disease. As they are already standard markers routinely used by pathologists, E-CADH, KRT7, KRT18, KRT19 are selected as epithelial markers, while vimentin (VIM) is selected as an indicator of mesenchymal phenotype. We analyse marker expression profiles and their relations with treatment responsiveness and prognosis of HGSC. This extensive analysis includes 18 HGSC cell lines, tissues from patient cohorts including the largest HGSC cohort in Canada, and publicly available datasets.

## 2. Results

### 2.1. HGSC Cell Lines and Tissues Display a High Level of Epithelial Marker Plasticity

**Figure 1:**
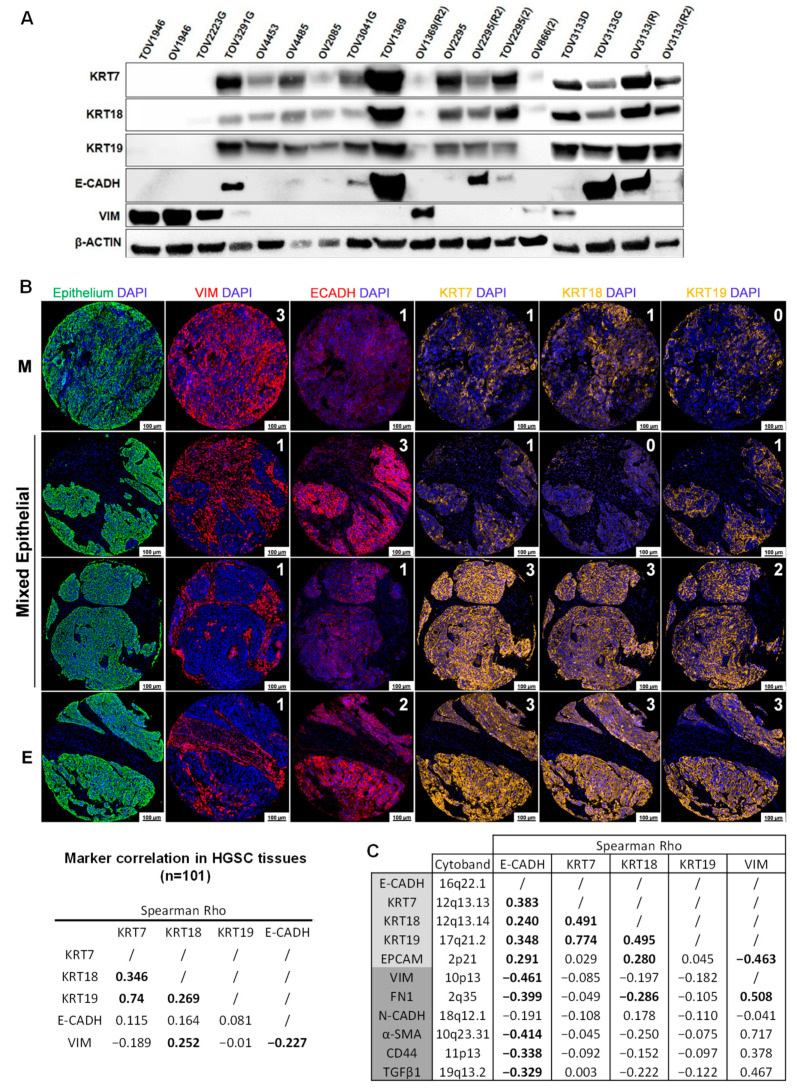
HGSC cell lines and tissues display a high variability in epithelial marker expression. (**A**) E-CADH, KRT7, KRT18, KRT19, and VIM protein expression was observed by Western blot in 18 HGSC cell lines. β-actin was used as normalization control. (**B**) KRT7, KRT18, KRT19, E-CADH, and VIM protein expression was observed by immunofluorescence in a TMA comprised of 101 HGSC tissues. Mean fluorescence intensity (MFI) of markers was quantified in the epithelium following digital image analysis. MFI were categorized into 4 groups of expression: 0 (negative), 1 (low), 2 (moderate), and 3 (high), using 25th, 50th and 75th percentiles, respectively, as cut-offs between groups. Spearman Rho correlations between marker expression are indicated in the table. M: Mesenchymal-like phenotype, E: Epithelial-like phenotype. (**C**) Spearman Rho correlations between KRT7, KRT18, KRT19, E-CADH, VIM, and EMT markers were obtained from protein expression measured by the CPTAC (mass spectrometry) on 274 HGSC cases extracted from TCGA (Ovarian Serous Cystadenocarcinoma dataset). Epithelial markers are highlighted in light grey and mesenchymal markers are highlighted in dark grey. Significant correlations are shown in bold (*p* < 0.05).

Protein level variability of epithelial and mesenchymal markers was analyzed in 18 HGSC cell lines (Figure 1A). TOV3291G, TOV1369, OV2295(R2), and TOV3133G cell lines showed an “epithelial-like” profile, as they expressed all epithelial markers but not VIM. Conversely, TOV1946, OV1946, and TOV2223G cell lines were defined as “mesenchymal-like”, due to a positive expression of VIM and no epithelial marker expression. Other cell lines showed a mixed profile, such as the OV4453, OV4485, OV2085, and OV2295 cell lines that expressed keratins while showing negative or weak E-CADH expression and various levels of VIM. We explored this marker plasticity in HGSC tissues from 101 patients represented in a Discovery TMA by immunofluorescence staining. MFI of markers was calculated in tumoral structures from each tissue using a robust and previously validated digital image analysis (Visiopharm^®^) [27,28]. Across patient tissues, we observed a great variability in marker intensity within epithelial structures, ranging from the mesenchymal-like to the epithelial-like phenotype, with most tissues exhibiting a mixed epithelial marker profile. Interestingly, E-CADH showed a negative correlation with VIM, whereas KRT7 and KRT19 did not correlate with this mesenchymal marker (Figure 1B). Using CPTAC protein expression from the TCGA Ovarian Serous Cystadenocarcinoma dataset (*n* = 274), we observed that protein expression of E-CADH was negatively correlated to markers of EMT, whereas KRT7 and 19 were not correlated to markers of EMT (Figure 1C). Our results highlight that HGSC tumors show a variability in epithelial marker expression across patients and suggest that KRT plasticity is independent from EMT status.

### 2.2. E-CADH, KRT7, and KRT19 Predict Patient Prognosis in the Discovery Cohort

**Figure 2:**
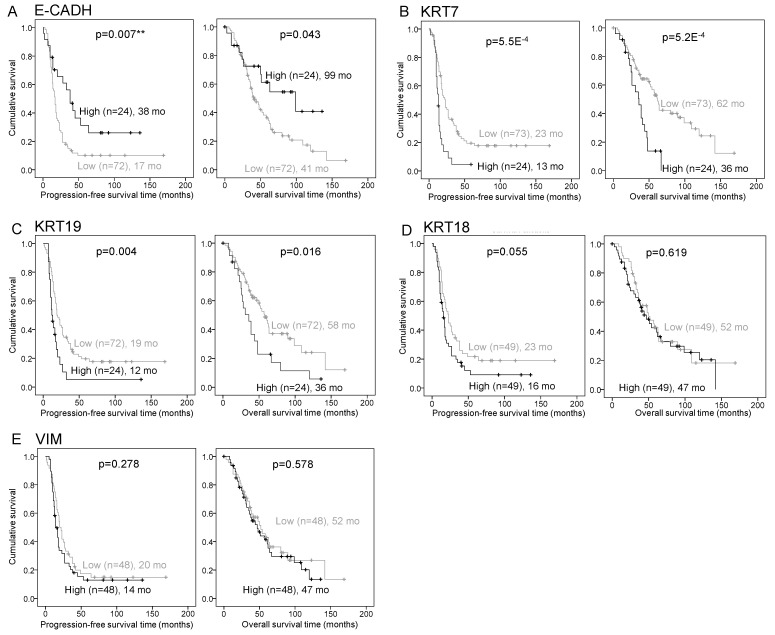
KRT7, KRT19, and E-CADH expression associate with prognosis in the HGSC Discovery cohort (*n* = 101). (**A**–**E**) Kaplan–Meier curves of E-CADH (**A**), KRT7 (**B**), KRT18 (**C**), KRT19 (**D**), and VIM (**E**) expression associations with progression-free survival (left) and overall survival (right). MFI measured expression of markers was dichotomized by the median for KRT18 and VIM and by the 75th percentile for E-CADH, KRT7, and KRT19, into groups of low and high expression. *p* values are indicated, ** = *p* < 0.01. Number of patients and estimated median survival months (mo) are indicated for each group.

Using the clinically annotated Discovery cohort (Table 1), we then sought to determine if epithelial marker plasticity could be an indicator of prognosis in patients with HGSC. KRT7 and KRT19 high expression was significantly associated with shorter progression-free survival (PFS) (*p* = 5.5 × 10^−4^ and *p* = 0.004, respectively) and shorter overall survival (OS) (*p* = 5.2 × 10^−4^ and *p* = 0.016, respectively), whereas E-CADH high expression was associated with longer PFS (*p* = 0.007) and longer OS (*p* = 0.043) (Figure 2A–C). KRT18 and VIM expression were not correlated with prognosis (Figure 2D,E). By univariate cox regression analysis, KRT7 and KRT19 expression showed a significant association with an unfavourable outcome, whereas E-CADH was associated with a favourable prognosis (Tables S2 and S3). Multivariate analysis indicated that KRT7 and KRT19 were independent prognosis biomarkers when adjusted for stage and residual disease (Tables S2 and S3).

### 2.3. E-CADH and KRT7 Combination Is the Best Prognosis Predictor in the Discovery Cohort

**Figure 3:**
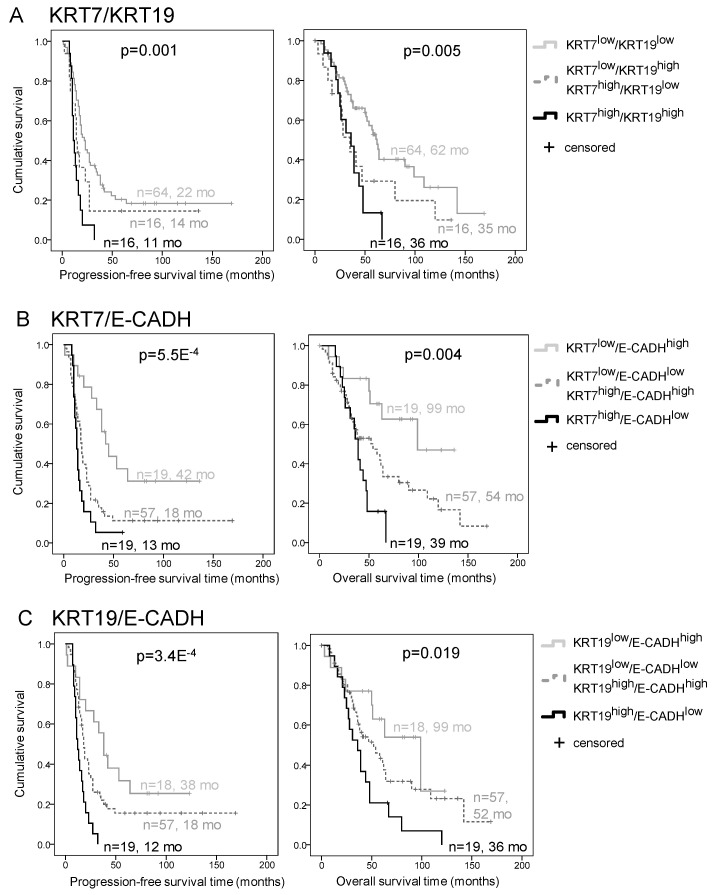
Combination of KRT7 and E-CADH expression improves the prediction of HGSC prognosis in the Discovery cohort (*n*-101). (A–C) Kaplan–Meier curves of KRT7/KRT19 (**A**), E-CADH-KRT7 (**B**), and KRT19/E-CADH (**C**) signature associations with progression-free survival (left) and overall survival (right). Expression of markers (MFI) was dichotomized by the 75th percentile into groups of low and high expression, and groups of patients were categorized as indicated in the legends. *p* values are indicated. Number of patients and estimated median survival months (mo) are indicated for each group.

Combinations of KRT7, KRT19, and E-CADH expression were evaluated to determine the most relevant signature to predict patient outcome. As KRT7 and KRT19 expression were highly correlated in patient HGSC from the Discovery cohort (Figure 2B), there was little added value in combining both markers compared to the individual markers regarding the prognosis evaluation (Figure 3A). However, the E-CADH-KRT7 and E-CADH-KRT19 combinations improved the level of prognosis prediction (Figure 3B,C, Table S2). The E-CADH-KRT7 combination was selected to pursue the study, since KRT7 alone showed greater levels of significance than KRT19 in evaluating patient prognosis (Figure 2, Tables S2 and S3). In addition, the E-CADH-KRT7 combination was a better independent prognosis predictor than E-CADH-KRT19 by multivariate analysis (Table S2).

### 2.4. E-CADH and KRT7 Signature Is an Independent Prognosis and Predictive Marker in the COEUR Cohort

**Figure 4:**
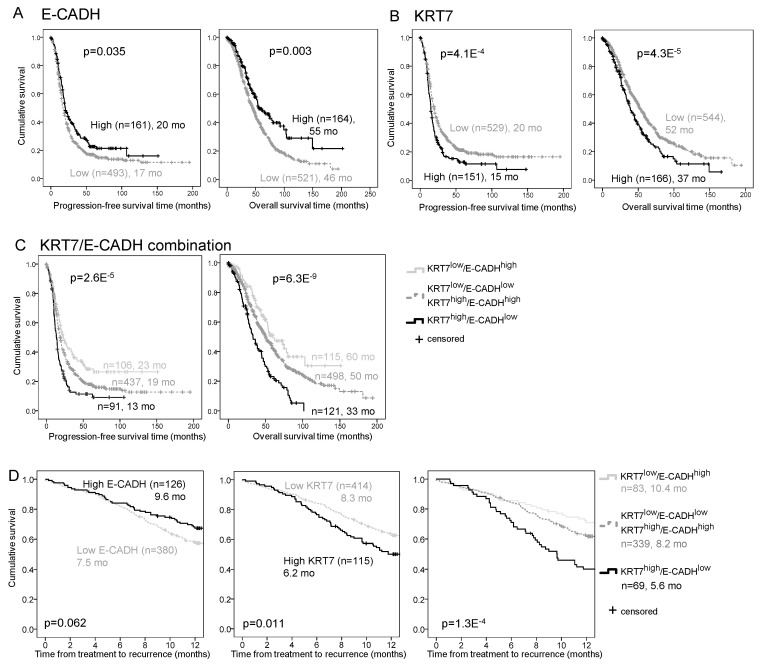
Combination of KRT7 and E-CADH expression predicts prognosis and treatment response in the HGSC COEUR cohort (*n* = 1031). (**A,B**) Kaplan–Meier curves of E-CADH (**A**), KRT7 (**B**), and E-CADH-KRT7 signature (**C**) associations with progression-free survival (left) and overall survival (right). Expression of markers (MFI) was dichotomized by the 75th percentile into groups of low and high expression, and groups of patients were categorized as indicated in the legends of E-CADH-KRT7 combination. Log ranks and *p* values are indicated. Number of patients and estimated median survival months (mo) are indicated for each group. (**D**) Kaplan–Meier curves of E-CADH (left), KRT7 (middle), and E-CADH-KRT7 signature (right) associations with 12 months’ time to recurrence after chemotherapy. *p* values are indicated. Number of patients and estimated 75th percentile months to recurrence are indicated for each group.

KRT7 and E-CADH expressions were probed in the pan-Canadian COEUR cohort TMA (*n* = 1158). After the selection of chemotherapy naïve patients with HGSC histotype, the analysis was restricted to 1031 patients (Table 1). E-CADH low expression and KRT7 high expression were associated with shorter PFS (*p* = 0.035 and *p* = 4.1 × 10^−4^, respectively) and shorter OS (*p* = 0.003 and *p* = 4.3 × 10^−5^, respectively) by Kaplan–Meier analysis (Figure 4A,B). Cox regression analyses confirmed that E-CADH and KRT7 were independent biomarkers of OS (*p* = 0.031 and *p* = 0.041, respectively) (Table S4). The combination of these markers improved the stratification of patients regarding PFS (*p* = 2.6 × 10^−5^) and OS (*p* = 6.3 × 10^−^^9^) by Kaplan–Meier analysis (Figure 4C) and improved the prediction of PFS (*p* = 0.020) and OS (*p* < 0.001) in multivariate analyses (Table S4). The ratio of KRT7/E-CADH levels showed a better association with progression status (Area Under the Curve (AUC) 0.602, *p* = 2.0 × 10^−4^) and survival status (AUC 0.609, *p* = 1.6 × 10^−^^6^) than each marker alone by Receiving Operating Characteristic (ROC) curve analysis (Figure S1). In addition to being predictive of all treatments (Figure S2), the E-CADH-KRT7 combination was also the best predictive marker of 12-month response to platinum + taxol-based chemotherapy (*p* = 1.3 × 10^−4^) by Kaplan–Meier analysis, compared to E-CADH (*p* = 0.062) and KRT7 (*p* = 0.011) alone, indicating that patients with high KRT7 and low E-CADH have a poorer response to the standard chemotherapy (Figure 4D).

### 2.5. E-CADH and KRT7 Signature Improves Patient Prognosis Stratification by Stage and Residual Disease in the COEUR Cohort

**Figure 5:**
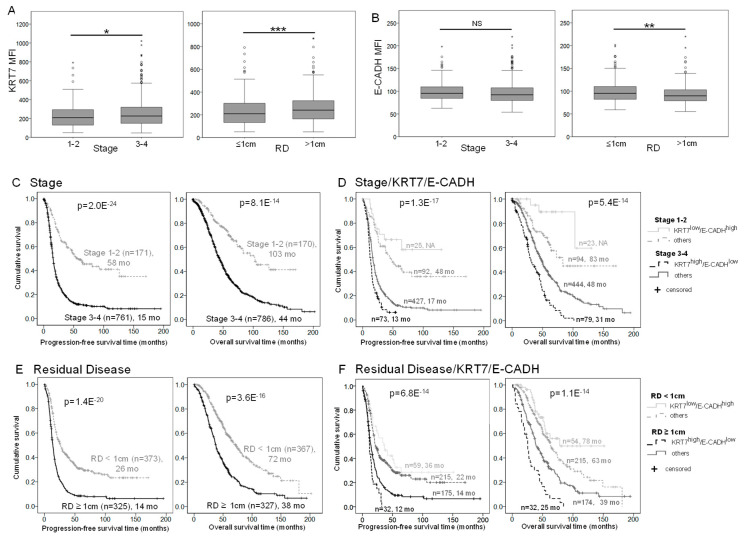
KRT7 and E-CADH combination improves prognosis evaluation of patients stratified by residual disease and stage. (**A**,**B**) KRT7 (**A**) and E-CADH (**B**) expression by early (1 and 2) or late (3 and 4) stage and by level of residual disease at surgery (low RD ≤ 1 cm or high RD > 1 cm). Boxes represent the interquartile (IQ) range and whiskers represent the highest and lowest values, which are less than 1.5 times the IQ range. Outliers are 1.5 times (circle) or 2 times (star) the IQ range. The non-parametric Mann-Whitney test was used to compare the means (* = *p* < 0.05; ** = *p* < 0.01; *** = *p* < 0.001). (**C**–**F**) Kaplan–Meier curves of stage (**C**), stage/E-CADH-KRT7 combination (**D**), RD (**E**), and RD/E-CADH-KRT7 combination (**F**) associations with progression-free survival (left) and overall survival (right). MFI measured expression of KRT7 and E-CADH was dichotomized by the 75th percentile into groups of low and high expression, and the groups of patients were categorized as indicated in the legends. *p* values are indicated. Number of patients and estimated median survival months (mo) are indicated for each group (NA = not applicable).

Association of the markers with clinical parameters of the COEUR cohort indicated that KRT7 levels were significantly elevated in tumors at late stages compared to tumors at early stages, and in tumors with high levels of residual disease (RD) compared to tumors with absence or low rates of RD after cytoreductive surgery (Figure 5A). Conversely, E-CADH expression was decreased in tumors with high levels of RD compared to those with low rate of RD after surgery (Figure 5B). This observation led us to evaluate the prognostic signature of the E-CADH-KRT7 combination in patients stratified by stage and RD, which were the strongest clinical parameters to evaluate HGSC patient prognosis (Figure 5C,E) in the COEUR cohort. The addition of KRT7 and E-CADH expression criteria enhanced the discrimination level of patients by estimated median survival months among the groups stratified by early/late tumor stages or by low/high RD rates (Figure 5D,F). By ROC curve analysis, the addition of KRT7/E-CADH levels to clinical parameters such as stage, RD, age, and/or BRCA mutation status systematically improved the performance to predict patient overall survival (Figure S3). Interestingly, using KRT7/E-CADH levels, stage and RD was highly predictive with an AUC 0.722 (*p* = 8.27 × 10^−^^14^, *n* = 473), and the addition of BRCA mutation status increased the performance to AUC 0.743 (*p* = 9.78 × 10^−^^10^, *n* = 231), thought it was on a more restricted number of patients.

### 2.6. KRT7 Is a Major Predictor of HGSC Patient Prognosis at the Gene Expression Level

E-CADH (CDH1) and KRT7 gene expression were then analysed in publicly available datasets. E-CADH expression was not correlated with prognosis at the gene expression level in the TCGA dataset or in the Kaplan–Meier plotter dataset (Figure S4A,B), corroborating the literature about E-CADH gene expression and ovarian cancer [29]. This observation can be explained by the weak correlation between E-CADH mRNA and its protein expression (Spearman Rho = 0.26) as observed in the TCGA dataset, suggesting that E-CADH mRNA expression does not reflect the level of E-CADH protein in HGSC tumors (Figure S4C).

Conversely, high KRT7 gene expression was significantly associated with poor patient prognosis in the Kaplan–Meier plotter dataset and in the CSIOVDB dataset comprised of 3431 OC patients classified by molecular subtypes [7] (Figure S5A,B). KRT7 showed a decreased mRNA expression and an elevated level of DNA methylation in the mesenchymal subtype compared to the epithelial-A and epithelial-B subtypes, indicating that KRT7 overexpression is not a feature of the mesenchymal profile (Figure S5C,D). Analysis of the TCGA dataset showed a high correlation between KRT7 mRNA and its protein expression in HGSC tumors (Spearman Rho = 0.71) (Figure S5E). When we focused on the Kaplan–Meier plotter dataset and categorized patients by stage or RD, addition of KRT7 mRNA expression improved the stratification of patient prognosis (Figure S6A). High KRT7 level was also predictive of a poorer 12-month response to treatments, particularly for the group of patients treated by platinum-based chemotherapy (Figure S6B). Together, our results indicate that KRT7 is a major HGSC prognostic biomarker at protein and gene expression levels and is predictive of a poorer response to chemotherapy.

### 2.7. KRT7 Is a Prognosis Biomarker of Breast, Gastric, and Non-Small-Cell Lung Carcinomas

Using the Kaplan–Meier plotter dataset, we analyzed KRT7 gene expression in breast, gastric, and non-small-cell lung carcinomas. In breast cancer, high KRT7 gene expression was associated with a poorer prognosis (Figure S7A). When we classified patients by breast cancer subtypes, we observed that higher KRT7 levels were associated with the poorest outcome in basal and HER2^+^ subtypes but not in the more differentiated luminal A and luminal B subtypes (Figure S8). Elevated KRT7 mRNA was also significantly associated with poorer outcomes in the intestinal subtype of gastric carcinoma (Figure S7B) and in lung adenocarcinoma, the most common subtype of non-small-cell lung cancer (Figure S7C). These last findings highlight the interest in evaluating the prognostic value of KRT7 in solid epithelial cancers.

## 3. Discussion

In our study, heterogeneous profiles of epithelial markers were observed in HGSC cell lines and tumor tissues. In the Discovery and the COEUR validation set, we showed that KRT7 expression is a strong and independent negative prognostic biomarker and that its combination with E-CADH expression further improved prognostic and treatment response prediction in HGSC patients. In recent years, researchers and clinicians have put much effort into finding new prognostic and predictive biomarkers of HGSC and only a few have been clinically validated [30,31,32]. A recent publication from Millstein et al. has proposed a 101 gene expression signature to predict high-grade serous ovarian cancer overall survival [33]. Performance of their signature including clinical parameters such as age and stage was an AUC of 0.75 for a five-year OS by ROC curve analysis. Here, our two-marker signature performance reached an AUC of 0.743 to predict the OS when associated with clinical parameters. Moreover, KRT7 is routinely used by gynecological pathologists to distinguish ovarian neoplasms from metastatic colonic adenocarcinoma [15,16]. E-CADH is commonly used in pathology to confirm epithelial cell lineage. Importantly, our results were validated using the same 75th percentile threshold for dichotomization across all studied cohorts for both markers and across TCGA and Kaplan–Meier plotter datasets for KRT7, indicating that this specific threshold does not rely on specific cohort data. Though our results were obtained by immunofluorescence, KRT7 and E-CADH expression analysis by immunohistochemistry are regularly conducted in pathology departments and this prognostic signature could be implemented in the clinical setting, at a lower cost than genomic analyses.

The ability of the E-CADH-KRT7 combination to discriminate patient prognosis with a higher reliability than KRT7 or E-CADH alone, probably lies in the fact that the two markers are involved in different pathways that participate in HGSC progression. E-CADH downregulation is associated with increased cell motility and cell invasion capacity and can be regulated by several EMT transcription factors including TWIST, SNAIL, SLUG, ZEB1, and TGF-β1, among others [11,34]. The combination of E-CADH and SNAIL protein expression was shown to be associated with ovarian cancer prognosis in a cohort of 174 patients [35]. Another study assessed the protein expression of E-CADH, N-CADH, P-cadherin, ZEB1, HMGA2, RAB25, CD24, NCAM, SOX11, and VIM in 100 tubo-ovarian serous carcinoma effusions and found a limited prognostic role of the markers alone or in combination [36]. Marker combinations from these last studies were less significant than the E-CADH-KRT7 signature in predicting patient prognosis, probably because of the redundancy of E-CADH and other markers’ involvement in EMT pathways. As expected, we observed a negative correlation between E-CADH and VIM in the Discovery cohort and between E-CADH and EMT markers in HGSC samples from the TCGA. In contrast, our results indicate that KRT7 protein expression is not associated with EMT markers or the mesenchymal OC subtype, suggesting that KRT7 upregulation is not a feature of EMT in HGSC.

The literature is scarce and mixed regarding KRT7 regulation in cancer. It was observed in a recent publication that KRT7 overexpression in ovarian cancer cell lines was associated with increased proliferation, migration and EMT marker expression through the regulation of the TGF-β/Smad2/3 [23]. These results obtained on HGSC cell lines differ from our findings which show that KRT7 expression is not correlated with EMT markers in HGSC tissues and emphasizes the need to more fully characterize KRT7 functions in HGSC progression. KRT7 regulation may be in part related to Forkhead box family members. In ovarian cancer cell line SKOV3, KRT5 and KRT7 were upregulated by Forkhead box M1 (FOXM1) and KRT5 and KRT7 deficiency prevented migration [24]. FOXM1 has been widely involved in cancer progression and several molecules targeting FOXM1 pathway are currently under investigation [37,38]. In esophageal squamous cell and in gastric carcinoma, where KRT7 overexpression is associated with a poor prognosis, KRT7 was transcriptionally upregulated by FOXA1 [20,39,40]. Other KRT7 regulation mechanisms involved the long non-coding RNA KRT7-AS that forms an RNA-RNA duplex with KRT7 and stabilizes KRT7 expression at the mRNA and the post-transcriptional levels. KRT7-AS promoted gastric and colorectal cancer cell progression by increasing KRT7 expression [41,42]. As these observations are disparate and were obtained from limited cell line models, there is a necessity to deepen the understanding of the mechanisms of regulation governing KRT7 expression. KRT7 regulation may involve several complex and tissue specific pathways that might represent interesting therapeutic targets.

Our analysis of KRT7 in publicly available cancer databases has highlighted that KRT7 gene expression is a prognostic marker of poor outcome in several cancer types including breast, non-small-cell lung, and gastric carcinomas. This last observation corroborates results from a recent publication where KRT7 overexpression was associated with poor prognosis in gastric cancer patients [21]. Other published works have indicated that elevated KRT7 is also associated with an unfavourable outcome in pancreatic cancer [43], esophageal squamous cell carcinoma [20] and colorectal carcinoma [22]. However, high KRT7 expression was associated with better OS in papillary renal cell carcinoma [44] and the KRT7/KRT19 expressing subtype was associated with better outcomes in clear cell renal cell carcinoma [45]. Together, these observations emphasize the major but complex and tissue-specific implications of KRT7 function in cancer progression.

Roles have been described for other KRTs than KRT7 in immune system and inflammation, DNA damage response and resistance to apoptosis, shear stress resistance during extravasation, or apico-basal polarization [10,46]. In addition, KRTs and notably KRT7, KRT18 and KRT19, are widely used to detect circulating tumor cells in the blood or detached tumor cells in ascites [46]. Soluble protein fragments of keratins, including KRT7, KRT8, KRT18 and KRT19, can be detected in the circulation of cancer patients and are used to monitor disease progression and patient prognostic in certain tumour types [46,47,48,49]. High level of KRT7 in serum of patients with non-small cell lung cancer complicated with superior vena cava syndrome was associated with a poor prognosis [47]. The utility of KRT7 as a liquid biopsy diagnostic, prognostic and predictive biomarker should be further evaluated in HGSC patients.

Our study was limited to several markers and the inclusion of other epithelial markers may be of interest to improve HGSC prognosis evaluation. Notably, EPCAM protein expression was associated with stemness, aggressive features and chemoresistance in ovarian cancer [50,51,52]. Potential KRT7 interaction with EPCAM and the relevance of adding EPCAM expression to the E-CADH-KRT7 signature should be further evaluated. In addition, the impact of epithelial plasticity and KRT7 functions on chemoresistance need to be explored, as we observed that patients with high KRT7 and low E-CADH levels had a poorer response to treatment and notably to platinum + taxol-based chemotherapy. Further studies are needed to understand the biological causes and significance of specific epithelial cell phenotypes in HGSC tumor progression and metastasis. We believe that the E-CADH-KRT7 combination is a very promising signature to predict HGSC patient prognosis and standard treatment response and could prove valuable in clinical decision making.

## 4. Materials and Methods

### 4.1. Patient Cohorts and Datasets

Ethics statement and patient cohorts for protein expression analysis.

Ethical approval (CER CHUM, REB Project Number: BD04.002, 13 November 2021) was obtained from the Centre hospitalier de l’Université de Montreal (CHUM) institutional ethics committee (Comité d’éthique de la recherche du CHUM).

HGSC specimens were collected during primary cytoreductive surgery of patients and subsequently formalin-fixed and paraffin-embedded (FFPE). Informed patient consent was obtained. HGSC tissue micro-arrays (TMAs) from the CHUM and the Terry Fox Research Institute (TFRI)-COEUR have previously been described [27,53,54,55]. Clinicopathological characteristics of the cohorts are summarized in Table 1. Inclusion criteria for the analysis were HGSC histopathology and chemotherapy naïve patients at surgery.

Two 0.6 mm tumor tissue punches from 101 patients were included in the Discovery TMA. Patients were recruited at the CHUM between 1993 and 2012. A gynecological pathologist (KR) assigned the histotype and tumor grade of tumor samples at the time of surgery, according to the International Federation of Gynecology and Obstetrics (FIGO).

The COEUR cohort included eight TMA blocks constructed from 1158 HGSC tumor samples. Patients were recruited between 1991 and 2017 from 10 tumor banks across Canada, including the CHUM [54]. Seventy-seven patient cases were common between the Discovery and the COEUR cohorts. Two 0.6 mm tumor tissue punches per patient were included in the COEUR TMA. Two gynaecologic-oncologic pathologists (MK and KR) performed a double central review of the FFPE TMA blocks with integrated use of diagnostic immunohistochemical markers [55].

### 4.2. Datasets for Gene Expression Analysis

#### 4.2.1. The Cancer Genome Atlas Dataset (TCGA)

E-CADH and KRT7 expression analysis was performed using mRNA expression (z-scores RNA Seq V2 RSEM, *n* = 307 samples) and proteomic data (z-scores mass spectrometry, *n* = 174 samples) from the TCGA Ovarian Serous Cystadenocarcinoma dataset (Firehose Legacy, *n* = 606). Proteomic data were obtained from the Clinical Proteomic Tumor Analysis Consortium (CPTAC), NCI/NIH on 1 November 2017: https://proteomics.cancer.gov/programs/cptac. Clinical data, CPTAC proteomic data and mRNA expression were downloaded from the cBioPortal for Cancer Genomics on November 2017: http://www.cbioportal.org [56].

#### 4.2.2. Kaplan–Meier Plotter Dataset

The Kaplan–Meier plotter is comprised of 1816 ovarian cancer patient data with a mean follow-up of 40 months (http://kmplot.com, accessed on 1 September 2019) [57]. The database was primarily set up using gene expression data and survival information of ovarian cancer patients downloaded from Gene Expression Omnibus (GEO) (*n* = 1251) and TCGA (*n* = 565) (Affymetrix HG-U133A, HG-U133A 2.0, and HG-U133 Plus 2.0 microarrays). Analysis was done on September 2019 with the JetSet best probe sets “201131_s_at” against E-CADH (*CDH1* gene) and “209016_s_at” against *KRT7* and was restricted to serous histology, grades 2 (*n* = 325) + 3 (*n* = 1024), all *TP53* status and all available datasets. Stage, debulking status, and chemotherapy regimens were variable parameters according to the analysis.

#### 4.2.3. Ovarian Cancer Database of the Cancer Science Institute Singapore (CSIOVDB)

CSIOVDB includes data on 3431 human ovarian carcinomas including carcinoma of the ovary (91.49%), fallopian tube, peritoneum, and metastasis to the ovary from GEO, ArrayExpress, TCGA, ExpO, and private/in-house data (http://csiovdb.mc.ntu.edu.tw/CSIOVDB.html, accessed on 1 May 2021). HGSC is the most highly represented carcinoma in CSIOVDB (73.75%) [29]. The database has 1516 and 1868 samples with progression-free survival (PFS) and overall survival (OS), respectively. Output of a gene query includes expression profiles in histological and molecular subtypes, survival correlations and integration with the DNA methylation from TCGA.

### 4.3. Immunofluorescence Staining

Immunostaining was performed on 4 µm TMA sections (Discovery and COEUR) using the Benchmark XT autostainer (Ventana Medical Systems, Roche, Rotkreuz, Switzerland). Antibody staining conditions were based on the manufacturer’s datasheet for each marker. Antigen retrieval was performed using Cell Conditioning 1 (Tris-EDTA buffer, pH 7.8, for KRT7, KRT18, KRT19, and VIM) or 2 (citrate buffer, pH 6.0, for E-CADH) (Ventana Medical Systems) and slides were then incubated for one hour with the primary mouse monoclonal antibodies against E-CADH (1/100, G10 sc-8426, Santa Cruz Biotechnology, Dallas, TX, USA), KRT7 (1/200, CLONE OV-TL 12/30, Thermo Scientific, Waltham, MA, USA), KRT18 (1/200, sc-6259, Santa Cruz Biotechnology), KRT19 (1/200, MS-198-P, Thermo Scientific) or VIM (1/200, sc6260, Santa Cruz Biotechnology). Epithelial cells were identified using a highly sensitive cocktail of rabbit monoclonal antibodies against KRT8/18 (FLEX, clone EP17/EP30, Dako, Mississauga, ON, Canada). Slides were incubated with secondary fluorescent antibodies for 45 min and cell nuclei were stained with DAPI. Slides were mounted with coverslips using Fluoromount medium (F4680, Millipore-Sigma, Oakville, ON, Canada).

### 4.4. Digital Image Analysis (DIA)

TMA slides were scanned with the VS-110 microscope using a 20X 0.75 NA objective and a resolution of 0.3225 µm (Olympus Canada Inc., Richmond Hill, ON, Canada) linked to an OlyVIA^®^ image viewer software (xvViewer.exe). Scanned images were imported into Visiopharm^®^ (VP) software (Hoersholm, Denmark), and fluorescent staining of the different markers were quantified by automated DIA as previously detailed [27]. VP algorithms used (1) DAPI staining to enable the delimitation of the “whole tissue” and (2) KRT8/18 staining to discriminate the regions of Interest (ROI) “epithelium” and “stroma” in each TMA punch. Marker expression was quantified in each image pixel of each ROI to calculate the mean fluorescence intensity (MFI) within the ROI. A visual review was also performed to exclude damaged tissue, necrotic, or red cell infiltrated sections.

### 4.5. High Grade Serous Carcinoma Cell Lines

All 18 cell lines used in our study were derived from HGSC solid tumors or peritoneal ascites and were described in publications from our group. Their characteristics are summarized in Table S1 [58,59,60]. Cell lines were cultivated at 37 °C in hypoxic condition of 7% O_2_, and 5% CO_2_ and grown in OSE medium (Wisent, St.-Bruno, QC, Canada) supplemented with 10% Foetal Bovine Serum, 0.5 μg/mL amphotericin B (Wisent), and 50 μg/mL gentamicin (Gibco^®^, Life Technologies Inc., Waltham, MA, USA).

### 4.6. Western Blot

The same antibodies were used for immunofluorescence and Western blot detection (referenced in the section “Immunofluorescence staining”), except for E-CADH. Total protein extracts (30 µg) were electrophoresed in 4–15% pre-cast gels (Bio-Rad, Mississauga, ON, Canada). Proteins were transferred onto PVDF membranes that were blocked in PBS with 5% milk. Membranes were then probed with primary antibodies in 5% milk PBS-Tween at the following dilutions: 1/10,000 for beta-actin, 1/1000 for KRT19, 1/2000 for KRT7 and KRT18, 1/500 for E-cadherin (24E10, #3195, Cell Signaling Technology Inc., Danvers, MA, USA) and vimentin. Protein expression was detected with HRP-conjugated secondary antibodies and visualized by enhanced chemiluminescence (ECL, Bio-Rad). Beta-actin was used as housekeeping gene loading control. As the 18 cell lines could not be loaded together on a same gel, 3291G protein extracts were used as a normalization control.

### 4.7. Statistics and Survival Analyses

Statistical analyses were conducted using IBM Statistics SPSS 23 and GraphPad Prism 5. Correlation studies between gene and protein expression were performed using non-parametric Spearman correlation. The non-parametric Mann-Whitney test was used to compare the mean between two groups of marker expression. A *p* value < 0.05 was considered statistically significant (* = *p* < 0.05; ** = *p* < 0.01; *** = *p* < 0.001).

For survival analyses, Kaplan–Meier, univariate, and multivariate Cox proportional hazard regression models and Receiving Operating Characteristic (ROC) curves were used. Multivariate analyses were performed in association with standard available prognostic indicators, including FIGO stages (1/2 vs. 3/4), RD at cytoreductive surgery (<1 cm vs. ≥1 cm/miliary), and age of patients at diagnosis (continuous values). *BRCA* mutation status was not considered for multivariate analyses, as this variable was not available for a large number of patients. Candidate biomarkers were systematically analysed by their continuous and dichotomized expression values by Cox regression analyses. Using the Discovery cohort, candidate expressions were dichotomized into groups of low and high expression by the 25th percentile, the median or the 75th percentile. For each candidate, the most significant cut-off was selected for the present study. KRT7 and E-CADH protein expression were dichotomized using the 75th percentile MFI cut-off in all the subsequent survival analyses, including patient categorization using the E-CADH-KRT7 combination. For AUC measures derived from ROC analyses, scores were a combination of the following dichotomized parameters: FIGO stage (0: 1/2 stages; 1: 3/4 stages), RD (0: <1 cm; 1: ≥1 cm/miliary), age (0: <65 years old; 1: ≥65 years old), *BRCA* mutation status (0: *BRCA1/2* mutation; 1: *BRCA1/2* wild type), and KRT7/E-CADH MFI expression ratio (0: <median ratio; 1: ≥median ratio).

## Figures and Tables

**Table 1:** Clinicopathological characteristics of the discovery and COEUR cohorts.

		Values (%)	
Variables		Discovery	COEUR
Number of patients	Total	101	1158
Age of patients at diagnosis	Median (years)	61.0	62.0
	Range (years)	34–81	26–91
Ovarian cancer histotype *	High grade serous	101 (100.0)	1093 (94.4)
	Low grade serous	/	31 (2.7)
	Endometrioid	/	14 (1.2)
	Clear cell	/	9 (0.8)
	Mucinous	/	2 (0.2)
	Unknown	/	9 (0.8)
*BRCA1/2* status	Wild-type	/	339 (29.3)
	*BRCA1* mutation	/	53 (4.5)
	*BRCA2* mutation	/	22 (1.9)
	*BRCA1*/*2* mutations^c^	/	3 (0.3)
	Unknown	/	741 (64.0)
Stage (FIGO)	1	4 (4.0)	77 (6.7)
	2	7 (6.9)	124 (10.7)
	3	72 (71.3)	801 (69.2)
	4	12 (11.9)	108 (9.30)
	Unknown	6 (5.9)	48 (4.10)
Residual disease	No residual disease	18 (17.8)	206 (17.8)
	Yes, size not specified	7 (6.9)	155 (13.4)
	≤1 cm	20 (19.8)	224 (19.3)
	1 cm–≤2 cm	22 (21.8)	81 (7.0)
	2 cm	26 (25.7)	171 (14.8)
	Miliary	3 (3.0)	34 (2.9)
	Unknown	5 (5.0)	287 (24.8)
Chemotherapy before surgery *	No	98 (97.0)	1093 (94.4)
	Yes	3 (3.0)	65 (5.6)
First line chemotherapy	Platinum ^b^ + taxol	76 (75.2)	901 (77.8)
	Platinum ^b^	2 (2.0)	59 (5.1)
	Taxol	2 (2.0)	3 (0.3)
	Others	21 (20.8)	98 (8.5)
	None	/	22 (1.9)
	Unknown	/	75 (6.5)
Overall survival time	Median (months)	48.0	36.1
	Range (months)	3–202	0–202
Progression free survival time	Median (months)	18.0	15.0
	Range (months)	1–202	0–195

* Patients with histopathology other than HGSC and patients with neoadjuvant chemotherapy were excluded from the analysis. ^a^ Number of years between sample collection and study. ^b^ Platinum includes cisplatin and/or carboplatin. ^c^ Patients with mutations on *BRCA1* and *BRCA2* genes.

## Data Availability

Data presented in this study are available on request from the corresponding author. The data are not publicly available due to privacy of patient clinical data.

## Supplementary Materials

Supplementary materials are available online at https://www.mdpi.com/article/10.3390/ijms22105325/s1.

## References

[1] Beirne J.P., McArt D.G., Roddy A., McDermott C., Ferris J., Buckley N.E., Coulter P., McCabe N., Eddie S.L., Dunne P.D. (2019). Defining the molecular evolution of extrauterine high grade serous carcinoma. Gynecol. Oncol..

[2] Labidi-Galy S.I., Papp E., Hallberg D., Niknafs N., Adleff V., Noe M., Bhattacharya R., Novak M., Jones S., Phallen J. (2017). High grade serous ovarian carcinomas originate in the fallopian tube. Nat. Commun..

[3] Lheureux S., Braunstein M., Oza A.M. (2019). Epithelial ovarian cancer: Evolution of management in the era of precision medicine. CA Cancer J. Clin..

[4] Talhouk A., George J., Wang C., Budden T., Tan T.Z., Chiu D.S., Kommoss S., Leong H.S., Chen S., Intermaggio M.P. (2020). Development and validation of the gene-expression Predictor of high-grade-serous Ovarian carcinoma molecular subTYPE (PrOTYPE). Clin. Cancer Res..

[5] Tothill R.W., Tinker A.V., George J., Brown R., Fox S.B., Lade S., Johnson D.S., Trivett M.K., Etemadmoghadam D., Locandro B. (2008). Novel molecular subtypes of serous and endometrioid ovarian cancer linked to clinical outcome. Clin. Cancer Res..

[6] The Cancer Genome Atlas Research Network (2011). Integrated genomic analyses of ovarian carcinoma. Nature.

[7] Tan T.Z., Miow Q.H., Huang R.Y., Wong M.K., Ye J., Lau J.A., Wu M.C., Bin Abdul Hadi L.H., Soong R., Choolani M. (2013). Functional genomics identifies five distinct molecular subtypes with clinical relevance and pathways for growth control in epithelial ovarian cancer. EMBO Mol. Med..

[8] Chen G.M., Kannan L., Geistlinger L., Kofia V., Safikhani Z., Gendoo D.M.A., Parmigiani G., Birrer M., Haibe-Kains B., Waldron L. (2018). Consensus on Molecular Subtypes of High-Grade Serous Ovarian Carcinoma. Clin. Cancer Res..

[9] Liao T.T., Yang M.H. (2017). Revisiting epithelial-mesenchymal transition in cancer metastasis: The connection between epithelial plasticity and stemness. Mol. Oncol..

[10] Sharma P., Alsharif S., Fallatah A., Chung B.M. (2019). Intermediate Filaments as Effectors of Cancer Development and Metastasis: A Focus on Keratins, Vimentin, and Nestin. Cells.

[11] Rosso M., Majem B., Devis L., Lapyckyj L., Besso M.J., Llaurado M., Abascal M.F., Matos M.L., Lanau L., Castellvi J. (2017). E-cadherin: A determinant molecule associated with ovarian cancer progression, dissemination and aggressiveness. PLoS ONE.

[12] Yu L., Hua X., Yang Y., Li K., Zhang Q., Yu L. (2017). An updated meta-analysis of the prognostic value of decreased E-cadherin expression in ovarian cancer. Oncotarget.

[13] Voutilainen K.A., Anttila M.A., Sillanpaa S.M., Ropponen K.M., Saarikoski S.V., Juhola M.T., Kosma V.M. (2006). Prognostic significance of E-cadherin-catenin complex in epithelial ovarian cancer. J. Clin. Pathol..

[14] Birchmeier W., Behrens J. (1994). Cadherin expression in carcinomas: Role in the formation of cell junctions and the prevention of invasiveness. Biochim. Biophys Acta.

[15] Ramalingam P. (2016). Morphologic, Immunophenotypic, and Molecular Features of Epithelial Ovarian Cancer. Oncology (Williston Park).

[16] Moll R., Zimbelmann R., Goldschmidt M.D., Keith M., Laufer J., Kasper M., Koch P.J., Franke W.W. (1993). The human gene encoding cytokeratin 20 and its expression during fetal development and in gastrointestinal carcinomas. Differentiation.

[17] Karantza V. (2011). Keratins in health and cancer: More than mere epithelial cell markers. Oncogene.

[18] Woelfle U., Sauter G., Santjer S., Brakenhoff R., Pantel K. (2004). Down-regulated expression of cytokeratin 18 promotes progression of human breast cancer. Clin. Cancer Res..

[19] Cen D., Chen J., Li Z., Zhao J., Cai X. (2017). Prognostic significance of cytokeratin 19 expression in pancreatic neuroendocrine tumor: A meta-analysis. PLoS ONE.

[20] Oue N., Noguchi T., Anami K., Kitano S., Sakamoto N., Sentani K., Uraoka N., Aoyagi K., Yoshida T., Sasaki H. (2012). Cytokeratin 7 is a predictive marker for survival in patients with esophageal squamous cell carcinoma. Ann. Surg. Oncol..

[21] Yang J. (2020). Identification of novel biomarkers, MUC5AC, MUC1, KRT7, GAPDH, CD44 for gastric cancer. Med. Oncol..

[22] Czapiewski P., Bobowicz M., Peksa R., Skrzypski M., Gorczynski A., Szczepanska-Michalska K., Korwat A., Jankowski M., Zegarski W., Szulgo-Paczkowska A. (2016). Keratin 7 expression in lymph node metastases but not in the primary tumour correlates with distant metastases and poor prognosis in colon carcinoma. Pol. J. Pathol..

[23] An Q., Liu T., Wang M.Y., Yang Y.J., Zhang Z.D., Liu Z.J., Yang B. (2020). KRT7 promotes epithelialmesenchymal transition in ovarian cancer via the TGFbeta/Smad2/3 signaling pathway. Oncol. Rep..

[24] Zhang Z., Tu K., Liu F., Liang M., Yu K., Wang Y., Luo Y., Yang B., Qin Y., He D. (2020). FoxM1 promotes the migration of ovarian cancer cell through KRT5 and KRT7. Gene.

[25] Ricciardelli C., Lokman N.A., Pyragius C.E., Ween M.P., Macpherson A.M., Ruszkiewicz A., Hoffmann P., Oehler M.K. (2017). Keratin 5 overexpression is associated with serous ovarian cancer recurrence and chemotherapy resistance. Oncotarget.

[26] Ouellet V., Provencher D.M., Maugard C.M., Le Page C., Ren F., Lussier C., Novak J., Ge B., Hudson T.J., Tonin P.N. (2005). Discrimination between serous low malignant potential and invasive epithelial ovarian tumors using molecular profiling. Oncogene.

[27] Communal L., Medrano M., Sircoulomb F., Paterson J., Kobel M., Rahimi K., Hoskins P., Tu D., Lheureux S., Oza A. (2020). Low junctional adhesion molecule-A expression is associated with an epithelial to mesenchymal transition and poorer outcomes in high-grade serous carcinoma of uterine adnexa. Mod. Pathol..

[28] Labouba I., Le Page C., Communal L., Kristessen T., You X., Peant B., Barres V., Gannon P.O., Mes-Masson A.M., Saad F. (2015). Potential Cross-Talk between Alternative and Classical NF-kappaB Pathways in Prostate Cancer Tissues as Measured by a Multi-Staining Immunofluorescence Co-Localization Assay. PLoS ONE.

[29] Tan T.Z., Yang H., Ye J., Low J., Choolani M., Tan D.S., Thiery J.P., Huang R.Y. (2015). CSIOVDB: A microarray gene expression database of epithelial ovarian cancer subtype. Oncotarget.

[30] Bolton K.L., Chenevix-Trench G., Goh C., Sadetzki S., Ramus S.J., Karlan B.Y., Lambrechts D., Despierre E., Barrowdale D., McGuffog L. (2012). Association between BRCA1 and BRCA2 mutations and survival in women with invasive epithelial ovarian cancer. JAMA.

[31] Sieh W., Kobel M., Longacre T.A., Bowtell D.D., deFazio A., Goodman M.T., Hogdall E., Deen S., Wentzensen N., Moysich K.B. (2013). Hormone-receptor expression and ovarian cancer survival: An Ovarian Tumor Tissue Analysis consortium study. Lancet Oncol..

[32] Goode E.L., Block M.S., Kalli K.R., Vierkant R.A., Chen W., Fogarty Z.C., Gentry-Maharaj A., Toloczko A., Hein A., Ovarian Tumor Tissue Analysis (OTTA) Consortium (2017). Dose-Response Association of CD8+ Tumor-Infiltrating Lymphocytes and Survival Time in High-Grade Serous Ovarian Cancer. JAMA Oncol..

[33] Millstein J., Budden T., Goode E.L., Anglesio M.S., Talhouk A., Intermaggio M.P., Leong H.S., Chen S., Elatre W., Gilks B. (2020). Prognostic gene expression signature for high-grade serous ovarian cancer. Ann. Oncol..

[34] Thiery J.P. (2002). Epithelial-mesenchymal transitions in tumour progression. Nat. Rev. Cancer.

[35] Takai M., Terai Y., Kawaguchi H., Ashihara K., Fujiwara S., Tanaka T., Tsunetoh S., Tanaka Y., Sasaki H., Kanemura M. (2014). The EMT (epithelial-mesenchymal-transition)-related protein expression indicates the metastatic status and prognosis in patients with ovarian cancer. J. Ovarian Res..

[36] Davidson B., Holth A., Hellesylt E., Tan T.Z., Huang R.Y., Trope C., Nesland J.M., Thiery J.P. (2015). The clinical role of epithelial-mesenchymal transition and stem cell markers in advanced-stage ovarian serous carcinoma effusions. Hum. Pathol..

[37] Gormally M.V., Dexheimer T.S., Marsico G., Sanders D.A., Lowe C., Matak-Vinkovic D., Michael S., Jadhav A., Rai G., Maloney D.J. (2014). Suppression of the FOXM1 transcriptional programme via novel small molecule inhibition. Nat. Commun..

[38] Halasi M., Hitchinson B., Shah B.N., Varaljai R., Khan I., Benevolenskaya E.V., Gaponenko V., Arbiser J.L., Gartel A.L. (2018). Honokiol is a FOXM1 antagonist. Cell Death Dis..

[39] Sano M., Aoyagi K., Takahashi H., Kawamura T., Mabuchi T., Igaki H., Tachimori Y., Kato H., Ochiai A., Honda H. (2010). Forkhead box A1 transcriptional pathway in KRT7-expressing esophageal squamous cell carcinomas with extensive lymph node metastasis. Int. J. Oncol..

[40] Liu B.L., Qin J.J., Shen W.Q., Liu C., Yang X.Y., Zhang X.N., Hu F., Liu G.M. (2019). FOXA1 promotes proliferation, migration and invasion by transcriptional activating KRT7 in human gastric cancer cells. J. Biol. Regul. Homeost. Agents.

[41] Chen S., Su T., Zhang Y., Lee A., He J., Ge Q., Wang L., Si J., Zhuo W., Wang L. (2020). Fusobacterium nucleatum promotes colorectal cancer metastasis by modulating KRT7-AS/KRT7. Gut Microbes.

[42] Huang B., Song J.H., Cheng Y., Abraham J.M., Ibrahim S., Sun Z., Ke X., Meltzer S.J. (2016). Long non-coding antisense RNA KRT7-AS is activated in gastric cancers and supports cancer cell progression by increasing KRT7 expression. Oncogene.

[43] Badea L., Herlea V., Dima S.O., Dumitrascu T., Popescu I. (2008). Combined gene expression analysis of whole-tissue and microdissected pancreatic ductal adenocarcinoma identifies genes specifically overexpressed in tumor epithelia. Hepatogastroenterology.

[44] Polifka I., Agaimy A., Herrmann E., Spath V., Trojan L., Stockle M., Becker F., Strobel P., Wulfing C., Schrader A.J. (2019). High proliferation rate and TNM stage but not histomorphological subtype are independent prognostic markers for overall survival in papillary renal cell carcinoma. Hum. Pathol..

[45] Mertz K.D., Demichelis F., Sboner A., Hirsch M.S., Dal Cin P., Struckmann K., Storz M., Scherrer S., Schmid D.M., Strebel R.T. (2008). Association of cytokeratin 7 and 19 expression with genomic stability and favorable prognosis in clear cell renal cell cancer. Int. J. Cancer.

[46] Werner S., Keller L., Pantel K. (2019). Epithelial keratins: Biology and implications as diagnostic markers for liquid biopsies. Mol. Aspects Med..

[47] Tong L., Xu H. (2019). Cytokeratin 7 and thyroid transcription factor—1 levels in patients with lung cancer complicated with superior vena cava syndrome and their correlation with clinicopathological characteristics. J. BUON.

[48] Jin C., Yang M., Han X., Chu H., Zhang Y., Lu M., Wang Z., Xu X., Liu W., Wang F. (2019). Evaluation of the value of preoperative CYFRA21-1 in the diagnosis and prognosis of epithelial ovarian cancer in conjunction with CA125. J. Ovarian Res..

[49] Pujol J.L., Molinier O., Ebert W., Daures J.P., Barlesi F., Buccheri G., Paesmans M., Quoix E., Moro-Sibilot D., Szturmowicz M. (2004). CYFRA 21-1 is a prognostic determinant in non-small-cell lung cancer: Results of a meta-analysis in 2063 patients. Br. J. Cancer.

[50] Garson K., Vanderhyden B.C. (2015). Epithelial ovarian cancer stem cells: Underlying complexity of a simple paradigm. Reproduction.

[51] Akhter M.Z., Sharawat S.K., Kumar V., Kochat V., Equbal Z., Ramakrishnan M., Kumar U., Mathur S., Kumar L., Mukhopadhyay A. (2018). Aggressive serous epithelial ovarian cancer is potentially propagated by EpCAM(+)CD45(+) phenotype. Oncogene.

[52] Tayama S., Motohara T., Narantuya D., Li C., Fujimoto K., Sakaguchi I., Tashiro H., Saya H., Nagano O., Katabuchi H. (2017). The impact of EpCAM expression on response to chemotherapy and clinical outcomes in patients with epithelial ovarian cancer. Oncotarget.

[53] Medrano M., Communal L., Brown K.R., Iwanicki M., Normand J., Paterson J., Sircoulomb F., Krzyzanowski P., Novak M., Doodnauth S.A. (2017). Interrogation of Functional Cell-Surface Markers Identifies CD151 Dependency in High-Grade Serous Ovarian Cancer. Cell Rep..

[54] Le Page C., Rahimi K., Kobel M., Tonin P.N., Meunier L., Portelance L., Bernard M., Nelson B.H., Bernardini M.Q., Bartlett J.M.S. (2018). Characteristics and outcome of the COEUR Canadian validation cohort for ovarian cancer biomarkers. BMC Cancer.

[55] Kobel M., Rahimi K., Rambau P.F., Naugler C., Le Page C., Meunier L., de Ladurantaye M., Lee S., Leung S., Goode E.L. (2016). An Immunohistochemical Algorithm for Ovarian Carcinoma Typing. Int. J. Gynecol. Pathol..

[56] Cerami E., Gao J., Dogrusoz U., Gross B.E., Sumer S.O., Aksoy B.A., Jacobsen A., Byrne C.J., Heuer M.L., Larsson E. (2012). The cBio cancer genomics portal: An open platform for exploring multidimensional cancer genomics data. Cancer Discov..

[57] Gyorffy B., Lanczky A., Szallasi Z. (2012). Implementing an online tool for genome-wide validation of survival-associated biomarkers in ovarian-cancer using microarray data from 1287 patients. Endocr. Relat. Cancer.

[58] Ouellet V., Zietarska M., Portelance L., Lafontaine J., Madore J., Puiffe M.L., Arcand S.L., Shen Z., Hebert J., Tonin P.N. (2008). Characterization of three new serous epithelial ovarian cancer cell lines. BMC Cancer.

[59] Letourneau I.J., Quinn M.C., Wang L.L., Portelance L., Caceres K.Y., Cyr L., Delvoye N., Meunier L., de Ladurantaye M., Shen Z. (2012). Derivation and characterization of matched cell lines from primary and recurrent serous ovarian cancer. BMC Cancer.

[60] Fleury H., Communal L., Carmona E., Portelance L., Arcand S.L., Rahimi K., Tonin P.N., Provencher D., Mes-Masson A.M. (2015). Novel high-grade serous epithelial ovarian cancer cell lines that reflect the molecular diversity of both the sporadic and hereditary disease. Genes Cancer.

