# Supplementary material for: A Keratin 7 and E-Cadherin Signature Is Highly Predictive of Tubo-Ovarian High-Grade Serous Carcinoma Prognosis
Source: Int J Mol Sci. 2021 May 18;22(10):5325. doi: 10.3390/ijms22105325 (PMC8158692; doi:10.3390/ijms22105325)
Supplement: Supplementary file 1 [file ijms-22-05325-s001.zip › ijms-1196827-supplementary.pdf]

A Keratin 7 and E-Cadherin Signature Is Highly Predictive of Tubo-Ovarian High-Grade Serous Carcinoma Prognosis

Laudine Communal [1,2], Noemi Roy [1,2], Maxime Cahuzac [1,2], Kurosh Rahimi [1,2,3], Martin Köbel [4], Diane M. Provencher [1,2,5] and Anne-Marie Mes-Masson [1,2,6,*]

1 Institut du cancer de Montréal, Montreal, QC H2X 0A9, Canada; laudine.communal@gmail.com (L.C.); noemi.roy@hotmail.com (N.R.); maxime.cahuzac@umontreal.ca (M.C.); kurosh.rahimi.chum@ssss.gouv.qc.ca (K.R.); diane.provencher.chum@ssss.gouv.qc.ca (D.M.P.)

2 Centre de recherche du Centre hospitalier de l'Université de Montréal (CRCHUM), Montreal, QC H2X 0A9, Canada

3 Department of Pathology, Centre Hospitalier de l'Université de Montréal (CHUM), Montreal, QC H3T 1J4, Canada

4 Department of Pathology and Laboratory Medicine, University of Calgary, Calgary, AB T2N 1N4, Canada; martin.kobel@cls.ab.ca

5 Division of Gynecologic Oncology, Université de Montréal, Montreal, QC H3T 1J4, Canada

6 Department of Medicine, Université de Montréal, Montreal, QC H3T 1J4, Canada

* Correspondence: anne-marie.mes-masson@umontreal.ca; Tel.: +1-514-890-8000 (ext. 25496)

Supplementary Data

Name	Histotype	Source	Site	Stage (FIGO)	Collection timepoint	Reference
TOV1946	HGSC	Dr A-M Mes-Masson	Solid Tumor	IIIC	Before treatment	Ouellet *et al*., 2008, BMC cancer
OV1946	HGSC	Dr A-M Mes-Masson	Peritoneal ascites	IIIC	Before treatment	
TOV2223G	HGSC	Dr A-M Mes-Masson	Solid Tumor	IIIC	Before treatment	
TOV1369	HGSC	Dr A-M Mes-Masson	Solid Tumor	IIIC	Before treatment	Letourneau *et al*., 2012, BMC cancer
OV1369(R2)	HGSC	Dr A-M Mes-Masson	Peritoneal ascites	IIIC	Post-treatment	
TOV2295(2)	HGSC	Dr A-M Mes-Masson	Solid Tumor	IIIC	Post-treatment	
OV2295	HGSC	Dr A-M Mes-Masson	Peritoneal ascites	IIIC	Before treatment	
OV2295(R2)	HGSC	Dr A-M Mes-Masson	Peritoneal ascites	IIIC	Post-treatment	
TOV3133G	HGSC	Dr A-M Mes-Masson	Solid Tumor	IIIC	Before treatment	
TOV3133D	HGSC	Dr A-M Mes-Masson	Solid Tumor	IIIC	Before treatment	
OV3133(R)	HGSC	Dr A-M Mes-Masson	Peritoneal ascites	IIIC	Post-treatment	
OV3133(R2)	HGSC	Dr A-M Mes-Masson	Peritoneal ascites	IIIC	Post-treatment	
TOV3291G	HGSC	Dr A-M Mes-Masson	Solid Tumor	III	Before treatment	Fleury *et al*., 2015, Genes & Cancer
OV866(2)	HGSC	Dr A-M Mes-Masson	Peritoneal ascites	IIIC	Post-treatment	
TOV3041G	HGSC	Dr A-M Mes-Masson	Solid Tumor	IV	Post-treatment	
OV4453	HGSC	Dr A-M Mes-Masson	Peritoneal ascites	IIIC	Before treatment	
OV4485	HGSC	Dr A-M Mes-Masson	Peritoneal ascites	IIIC	Post-treatment	
OV2085	HGSC	Dr A-M Mes-Masson	Peritoneal ascites	IIIC	Post-treatment	/

FIGO: International Federation of Gynecology and Obstetrics

Table S1: Characteristics of HGSC cell lines

Univariate analysis	Progression-free survival				Overall survival			
	Hazard Ratio	95% CI		P value	Hazard Ratio	95% CI		P value
Variable		Inf	Sup			Inf	Sup	
**Residual disease[+]**	2.584	1.575	4.238	<0.001	3.989	2.011	7.912	<0.001
**Stage[++]**	1.884	0.904	3.925	0.091	2.830	0.888	9.022	0.079
**Age at diagnosis**	0.995	0.974	1.016	0.652	1.003	0.978	1.028	0.833
**KRT7 (low vs high)**	2.357	1.414	3.931	0.001	2.590	1.481	4.530	<0.001
**KRT18 (low vs high)**	1.517	0.978	2.354	0.063	1.134	0.689	1.866	0.621
**KRT19 (low vs high)**	2.045	1.224	3.416	0.006	1.910	1.117	3.268	0.018
**E-CADH (low vs high)**	0.478	0.273	0.835	0.009	0.503	0.254	0.996	0.049
**VIM (low vs high)**	1.268	0.817	1.969	0.290	1.153	0.697	1.909	0.580
**KRT7-KRT19 comb**	1.695	1.259	2.281	<0.001	1.629	1.203	2.208	0.002
**E-CADH-KRT7 comb**	1.948	1.374	2.760	<0.001	1.986	1.322	2.985	<0.001
**E-CADH-KRT19 comb**	2.015	1.376	2.952	<0.001	1.745	1.165	2.615	0.007

Multivariate analysis	Progression-free survival				Overall survival			
	Hazard Ratio	95% CI		P value	Hazard Ratio	95% CI		P value
Variable		Inf	Sup			Inf	Sup	
**Residual disease[+]**	2.798	1.554	5.041	<0.001	4.949	2.102	11.653	<0.001
**Stage[++]**	0.948	0.415	2.165	0.900	0.768	0.205	2.880	0.695
**KRT7 (low vs high)**	2.081	1.223	3.541	0.007	2.371	1.319	4.262	0.004
**Residual disease[+]**	3.213	1.756	5.878	<0.001	5.935	2.447	14.400	<0.001
**Stage[++]**	0.786	0.331	1.867	0.585	0.606	0.152	2.414	0.477
**KRT19 (low vs high)**	2.060	1.202	3.531	0.009	2.042	1.159	3.596	0.013
**Residual disease[+]**	2.816	1.554	5.104	<0.001	4.943	2.064	11.839	<0.001
**Stage[++]**	0.817	0.349	1.913	0.642	0.703	0.180	2.742	0.612
**E-CADH (low vs high)**	0.566	0.306	1.046	0.069	0.619	0.285	1.344	0.225
**Residual disease[+]**	2.637	1.462	4.756	0.001	4.636	1.974	10.887	<0.001
**Stage[++]**	0.826	0.362	1.885	0.650	0.673	0.180	2.522	0.557
**E-CADH-KRT7 comb**	1.766	1.208	2.581	0.003	1.856	1.178	2.925	0.008
**Residual disease[+]**	2.832	1.554	5.162	<0.001	5.267	2.201	12.603	<0.001
**Stage[++]**	0.695	0.292	1.655	0.411	0.580	0.146	2.306	0.439
**E-CADH-KRT19 comb**	1.853	1.214	2.828	0.004	1.672	1.053	2.653	0.029

CI=confidence interval; Inf=inferior; Sup=superior, comb=combination

KRT18 and VIM expression and E-CADH, KRT7 and KRT19 expression were dichotomized into low and high expression by the median and the 75th percentile of continuous MFI values, respectively.

[+] Residual disease variable was dichotomized into absence/low rates (<1cm) and higher rates (≥1cm) of residual disease.

[++] Stage variable was dichotomized into early (FIGO stages 1 and 2) and advanced stages (FIGO stages 3 and 4).

Table S2: Cox univariate and multivariate analysis in the Discovery cohort

Univariate analysis	Progression-free survival				Overall survival			
	Hazard Ratio	95% CI		P value	Hazard Ratio	95% CI		P value
Variable		Inf	Sup			Inf	Sup	
**Residual disease[+]**	2.584	1.575	4.238	<0.001	3.989	2.011	7.912	<0.001
**Stage[++]**	1.884	0.904	3.925	0.091	2.830	0.888	9.022	0.079
**Age at diagnosis**	0.995	0.974	1.016	0.652	1.003	0.978	1.028	0.833
**KRT7 (continuous values)**	1.001	1.000	1.002	0.001	1.001	1.000	1.002	<0.001
**KRT18 (continuous values)**	1.000	1.000	1.001	0.085	1.000	0.999	1.000	0.716
**KRT19 (continuous values)**	1.001	1.000	1.002	0.014	1.001	1.000	1.002	0.009
**E-CADH (continuous values)**	0.988	0.973	1.003	0.112	0.984	0.965	1.003	0.090
**VIM (continuous values)**	1.000	0.999	1.001	0.724	1.000	0.998	1.001	0.907

Multivariate analysis	Progression-free survival				Overall survival			
	Hazard Ratio	95% CI		P value	Hazard Ratio	95% CI		P value
Variable		Inf	Sup			Inf	Sup	
**Residual disease[+]**	2.742	1.511	4.975	<0.001	5.106	2.143	12.164	<0.001
**Stage[++]**	0.872	0.377	2.018	0.749	0.663	0.173	2.546	0.550
**KRT7 (continuous values)**	1.001	1.000	1.002	0.006	1.001	1.000	1.002	0.003
**Residual disease[+]**	3.211	1.759	5.859	<0.001	6.066	2.516	14.624	<0.001
**Stage[++]**	0.865	0.371	2.014	0.737	0.624	0.161	2.422	0.495
**KRT19 (continuous values)**	1.001	1.000	1.002	0.017	1.001	1.000	1.002	0.007
**Residual disease[+]**	3.026	1.666	5.498	<0.001	5.285	2.202	12.686	<0.001
**Stage[++]**	0.882	0.377	2.061	0.772	0.729	0.187	2.832	0.648
**E-CADH (continuous values)**	0.990	0.975	1.004	0.154	0.985	0.967	1.004	0.129

CI=confidence interval; Inf=inferior; Sup=superior

[+] Residual disease variable was dichotomized into absence/low rates of residual disease (<1cm) and higher rates of residual disease (≥1cm).

[++] Stage variable was dichotomized into early (FIGO stages 1 and 2) and advanced stages (FIGO stages 3 and 4).

Table S3: Cox univariate and multivariate analysis of marker continuous values in the Discoverycohort.

Univariate analysis	Progression-free survival				Overall survival			
	Hazard Ratio	95% CI		P value	Hazard Ratio	95% CI		P value
**Variable**		Inf	Sup			Inf	Sup	
**Residual disease[+]**	2.224	1.871	2.644	<0.001	2.200	1.811	2.672	<0.001
**Stage[++]**	3.152	2.498	3.977	<0.001	2.749	2.084	3.626	<0.001
**Age at diagnosis**	1.004	0.997	1.010	0.247	1.019	1.011	1.026	<0.001
**Chemotherapy treatment[+++]**	1.484	1.226	1.794	<0.001	1.552	1.254	1.920	<0.001
**KRT7 (continuous value)**	1.001	1.001	1.002	<0.001	1.002	1.001	1.002	<0.001
**KRT7 (low vs high)**	1.435	1.173	1.755	<0.001	1.572	1.263	1.955	<0.001
**E-CADH (continuous value)**	0.996	0.992	1.000	0.059	0.992	0.987	0.997	<0.001
**E-CADH (low vs high)**	0.801	0.650	0.986	0.036	0.687	0.535	0.883	0.003
**E-CADH-KRT7 comb**					1.701	1.401	2.065	<0.001
E-CADH^{high}-KRT7^{low}	Reference			<0.001	Reference			<0.001
E-CADH^{low}-KRT7^{low} and E-CADH^{high}-KRT7^{high}	1.335	1.034	1.724	0.027	1.332	0.971	1.826	0.075
E-CADH^{low}-KRT7^{high}	2.079	1.505	2.872	<0.001	2.674	1.848	3.869	<0.001

Multivariate analysis	Progression-free survival				Overall survival			
	Hazard Ratio	95% CI		P value	Hazard Ratio	95% CI		P value
**Variable**		Inf	Sup			Inf	Sup	
**Residual disease[+]**	1.659	1.345	2.046	<0.001	1.666	1.309	2.121	<0.001
**Stage[++]**	2.657	1.872	3.772	<0.001	2.459	1.580	3.827	<0.001
**Age at diagnosis**	/	/	/	/	1.010	0.999	1.021	0.074
**Chemotherapy treatment[+++]**	1.390	1.085	1.782	<0.009	1.373	1.036	1.821	0.028
**KRT7 (low vs high)**	1.216	0.952	1.553	0.117	1.325	1.012	1.735	0.041
**Residual disease[+]**	1.608	1.301	1.988	<0.001	1.633	1.275	2.093	<0.001
**Stage[++]**	2.740	1.912	3.925	<0.001	2.448	1.555	3.855	<0.001
**Age at diagnosis**	/	/	/	/	1.009	0.998	1.020	0.101
**Chemotherapy treatment[+++]**	1.433	1.118	1.836	0.005	1.411	1.062	1.875	0.018
**E-CADH (low vs high)**	0.852	0.668	1.088	0.199	0.718	0.531	0.971	0.031
**Residual disease[+]**	1.620	1.302	2.015	<0.001	1.640	1.269	2.119	<0.001
**Stage[++]**	2.642	1.842	3.791	<0.001	2.367	1.501	3.733	<0.001
**Age at diagnosis**	/	/	/	/	1.008	0.997	1.020	0.147
**Chemotherapy treatment[+++]**	1.384	1.072	1.786	0.013	1.348	1.007	1.804	0.044
**E-CADH-KRT7 comb**								
E-CADH^{high}-KRT7^{low}	Reference			0.020	Reference			<0.001
E-CADH^{low}-KRT7^{low} and E-CADH^{high}-KRT7^{high}	1.145	0.852	1.539	0.369	0.991	0.687	1.431	0.963
E-CADH^{low}-KRT7^{high}	1.657	1.134	2.421	0.009	2.088	1.353	3.222	<0.001

CI=confidence interval; Inf=inferior; Sup=superior; comb=combination.

E-CADH and KRT7 low and high groups of expression were dichotomized by the 75th percentile of continuous MFI values.

[+] Residual disease variable was dichotomized into absence/low rates (<1cm) versus higher rates (≥1cm) of residual disease

[++] Stage variable was dichotomized into early (FIGO stages 1 and 2) versus advanced stages (FIGO stages 3 and 4).

[+++] Chemotherapy was dichotomized into platinum+taxane treatment versus other treatment (including no treatment) groups.

Table S4: Cox univariate and multivariate analysis in the COEUR cohort.

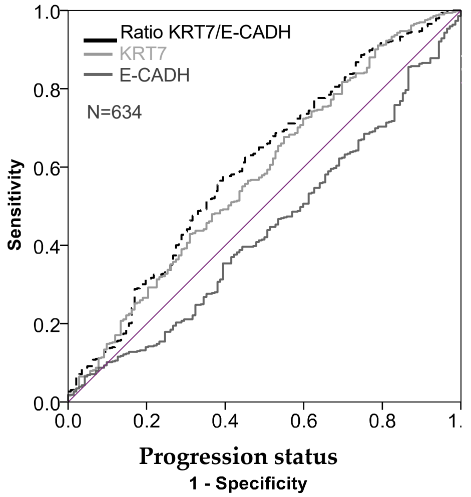

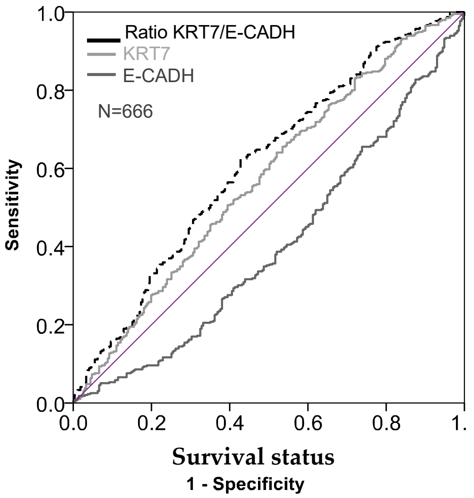

Test Result Variables	AUC	Std. Error[a]	p value[b]	Asymptotic 95% Confidence Interval	
				Lower Bound	Upper Bound
KRT7	0.581	0.028	0.003	0.526	0.635
E-CADH	0.440	0.027	0.030	0.388	0.493
Ratio KRT7/E-CADH	0.602	0.028	1.99E^{-04}	0.548	0.656

a. Under the nonparametric assumption

b. Null hypothesis: true area = 0.5

Test Result Variables	AUC	Std. Error[a]	p value[b]	Asymptotic 95% Confidence Interval	
				Lower Bound	Upper Bound
KRT7	0.572	0.023	0.002	0.527	0.616
E-CADH	0.402	0.022	1.69E^{-05}	0.358	0.446
Ratio KRT7/E-CADH	0.609	0.022	1.55E^{-06}	0.566	0.653

a. Under the nonparametric assumption

b. Null hypothesis: true area = 0.5

Figure S1: KRT7 and E-CADH expression associate with the progression and the survival status in the COEUR cohort. Receiving Operating Characteristic (ROC) curves of KRT7 and E-CADH expression according to the progression (left) and the survival status (right). The ratio KRT7/E-CADH was calculated by dividing KRT7 MFI value by E-CADH MFI value for each patient. Area under the curve (AUC), standard error (Std. Error), asymptotic significance (p value) and95% confidence interval are indicated.

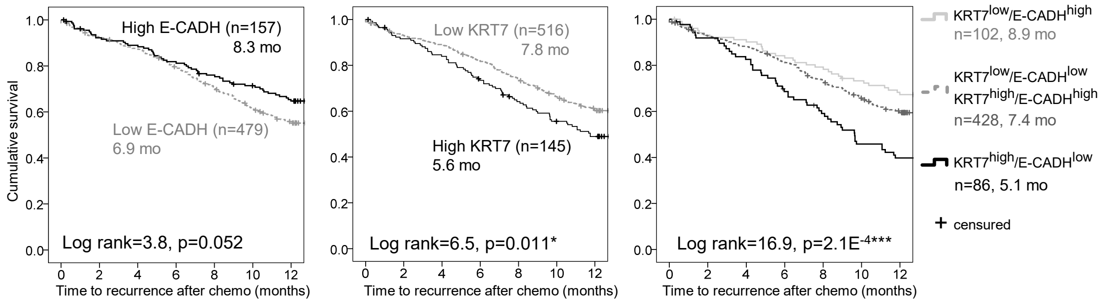

Figure S2: Combination of KRT7 and E-CADH expression predicts treatment response in the HGSC COEUR cohort. Kaplan-Meier curves of E-CADH (left), KRT7 (middle) and KRT7/E-CADH signature (right) associations with 12 months' time to recurrence after treatment, independently of the type of administered chemotherapy. Log ranks and p values are indicated. Number of patients and estimated 75th percentile months to recurrence are indicated for each group.

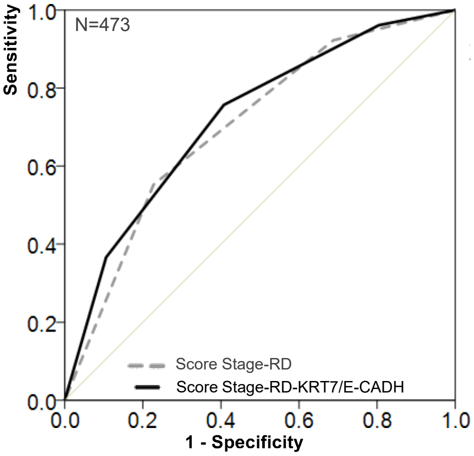

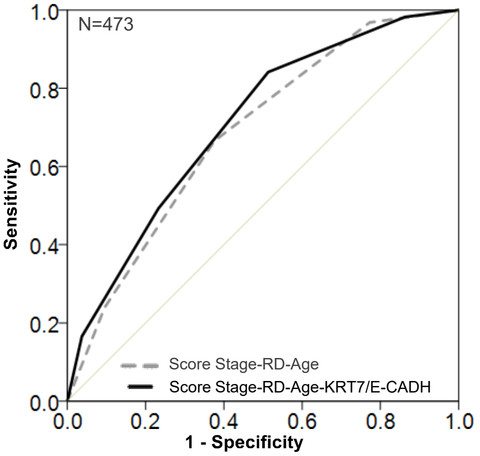

Area Under the Curve

Test Result Variables	AUC	Std. Error[a]	p value[b]	Asymptotic 95% Confidence Interval	
				Lower Bound	Upper Bound
Score Stage-RD	0.703	0.025	$8.27E^{-14}$	0.654	0.751
Score Stage-RD-KRT7/E-CADH	0.722	0.024	$3.05E^{-16}$	0.675	0.768

a. Under the nonparametric assumption

b. Null hypothesis: true area = 0.5

Area Under the Curve

Test Result Variables	Area	Std. Error[a]	p value[b]	Asymptotic 95% Confidence Interval	
				Lower Bound	Upper Bound
Score Stage-RD-Age	0.686	0.025	$6.43E^{-12}$	0.637	0.736
Score Stage-RD-Age-KRT7/E-CADH	0.709	0.024	$1.15E^{-14}$	0.662	0.757

a. Under the nonparametric assumption

b. Null hypothesis: true area = 0.5

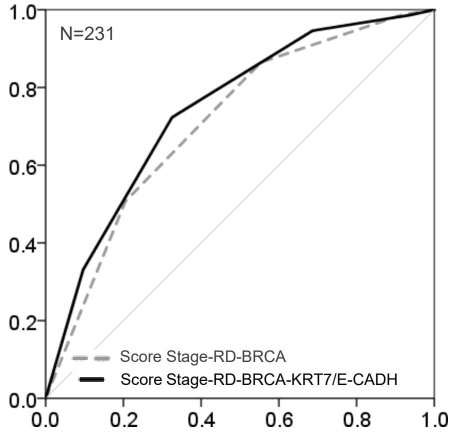

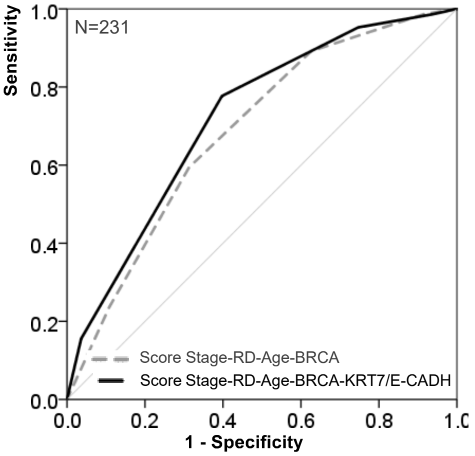

Test Result Variables	AUC	Std. Error[a]	p value[b]	Asymptotic 95% Confidence Interval	
				Lower Bound	Upper Bound
Score Stage-RD-BRCA	.710	.036	$1.22E^{-7}$	.639	.781
Score Stage-RD-BRCA-KRT7/E-CADH	.743	.034	$9.78.E^{-10}$	.675	.810

a. Under the nonparametric assumption

b. Null hypothesis: true area = 0.5

Test Result Variables	AUC	Std. Error[a]	p value[b]	Asymptotic 95% Confidence Interval	
				Lower Bound	Upper Bound
Score Stage-RD-Age-BRCA	.686	.037	$2.72E^{-6}$	.613	.759
Score Stage-RD-Age-BRCA-KRT7/E-CADH	.720	.036	$2.83E^{-8}$	.650	.790

a. Under the nonparametric assumption

b. Null hypothesis: true area = 0.5

Figure S3: KRT7 and E-CADH signature improve the performance of clinical parameters to predict overall survival in the COEUR cohort. Receiving Operating Characteristic (ROC) curves of patient scores including clinical parameters such as stage, RD, age, *BRCA* mutation status and/or KRT7/E-CADH expression ratio, as indicated, according to the survival status. The ratio KRT7/E-CADH was calculated by dividing KRT7 MFI value by E-CADH MFI value for each patient. Area under the curve (AUC), standard error (Std. Error), asymptotic significance (p value) and 95% confidence interval are indicated.

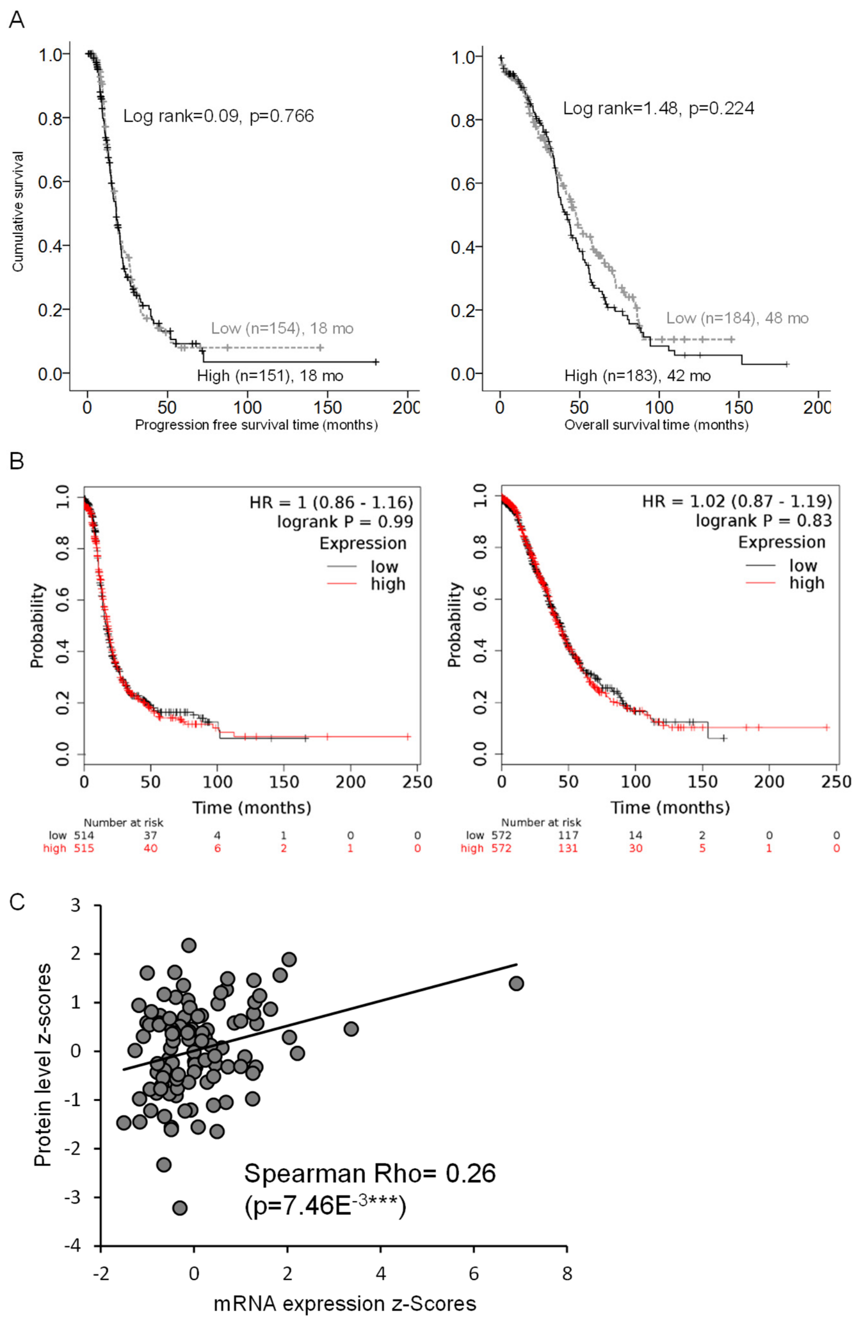

Figure S4: *E-CADH* gene expression does not correlate with prognosis in public datasets. A-B. Kaplan- Meier curves of *E-CADH* gene expression association with progression-free survival (left) and overall survival (right) in TCGA (**A**) and Kaplan-Meier Plotter (**B**) HGSC datasets. Gene expression of *E-CADH* was dichotomized into groups of low and high expression by the median cut-off in TCGA and Kaplan-Meier Plotter datasets. Log rank p values are indicated. Number of patients and estimated median number of survival months are indicated for each group. Hazard ratio (HR) and confidence intervals are reported for Kaplan-Meier plotter dataset. **C**. Correlation of E-CADH protein level z-scores from CPTAC and *E-CADH* mRNA z-scores (RNA Seq V2 RSEM) in overlapping cases (n=174) retrieved from the TCGA database. Spearman Rho and p value are indicated.

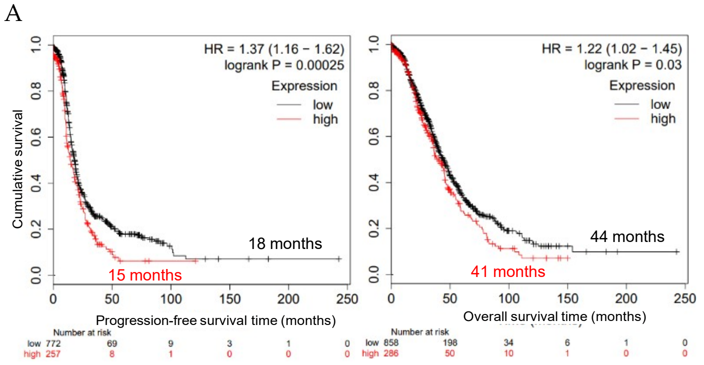

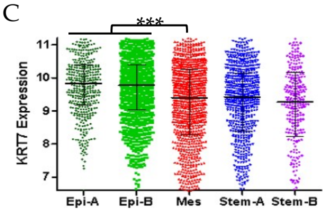

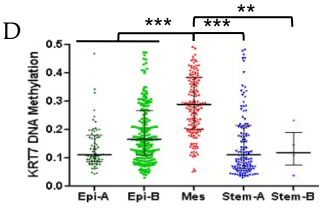

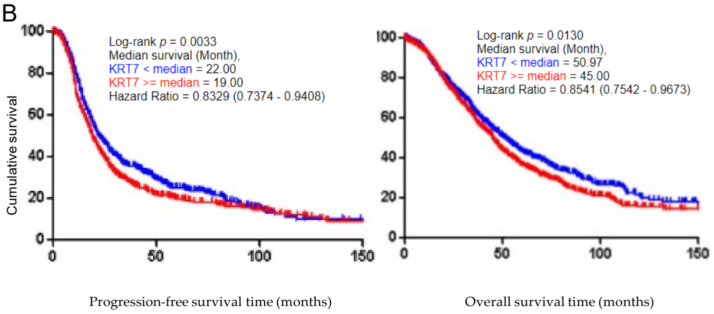

Multivariate Analysis - Disease-Free Survival

Factor	Cox Coefficient	*p*-value
Stage	1.266701	0.000000
Grade	0.278811	0.415013
Surgical Debulking	0.265763	0.004638
Histology	1.072631	0.000316
Age	0.180752	0.039302
KRT7	0.239176	0.006466

Multivariate Analysis - Overall Survival

Factor	Cox Coefficient	*p*-value
Stage	1.380306	0.000019
Grade	0.608844	0.113381
Surgical Debulking	0.204324	0.031283
Histology	1.282078	0.011034
Age	0.159988	0.078696
KRT7	0.267434	0.003439

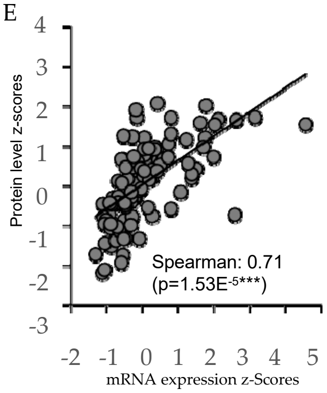

Figure S5: *KRT7* is associated with HGSC poor prognosis in gene expression datasets. **A-B**. Kaplan-Meier curves of *KRT7* gene expression association with progression-free survival (left) and overall survival (right) in Kaplan-Meier Plotter (**A**) and CSIOVDB (**B**) datasets. Gene expression of *KRT7* was dichotomized into groups of low and high expression by the 75th percentile cut-off in Kaplan-Meier Plotter dataset and by the reported median cut-off in CSIOVDB web interface. Log rank p values are indicated. Number of patients and estimated median survival months are indicated for each group. Hazard ratio (HR) and confidence intervals are reported for Kaplan-Meier plotter dataset. Cox multivariate analysis is reported for CSIOVDB dataset. **C-D**. *KRT7* gene expression (D) and *KRT7* gene methylation from TCGA by CSIOVDB molecular subtypes of ovarian carcinoma (n=3,431). Epi-A, Epi-B, Mes, Stem-A and Stem-B denote epithelial-A, epithelial-B, mesenchymal, stem-like-A and stem-like-B subtypes, respectively. **E**. Correlation of KRT7 protein level z-scores from CPTAC and *KRT7* mRNA z-scores (RNA Seq V2 RSEM) in overlapping cases (n=174) retrieved from the TCGA database. Spearman Rho and p values are indicated.

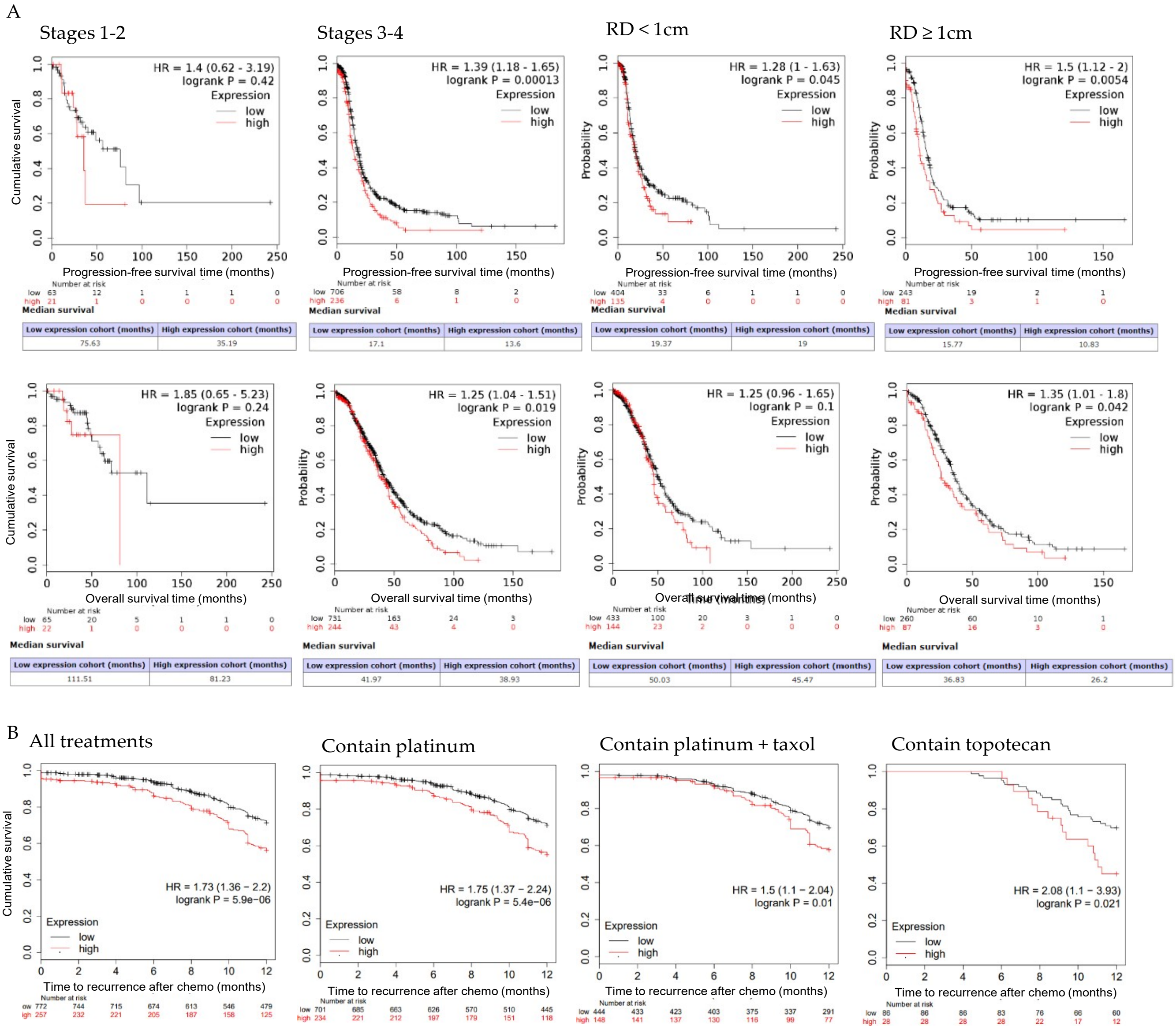

Figure S6: *KRT7* gene expression improves prognosis evaluation of patients stratified by stage and residual disease and predicts chemotherapy responses. Kaplan-Meier curves of *KRT7* gene expression associations with reported clinical parameters in Kaplan-Meier plotter ovarian cancer dataset. **A.** *KRT7* association with progression-free survival (up) and overall survival (below) in early stages (FIGO stages 1 and 2), late stages (FIGO stages 3 and 4), low residual disease (RD < 1cm) and high residual disease (RD ≥ 1cm) patient groups. **B.** *KRT7* association with recurrence time after chemotherapy in all treatments, platinum-based, platinum + taxol-based and topotecan-based treatment groups of patients. Gene expression of *KRT7* was dichotomized by the 75th percentile into groups of low and high expression. Log rank p values, hazard ratio (HR) and confidence intervals are indicated. Number of patients and estimated median number of survival months are reported for each group.

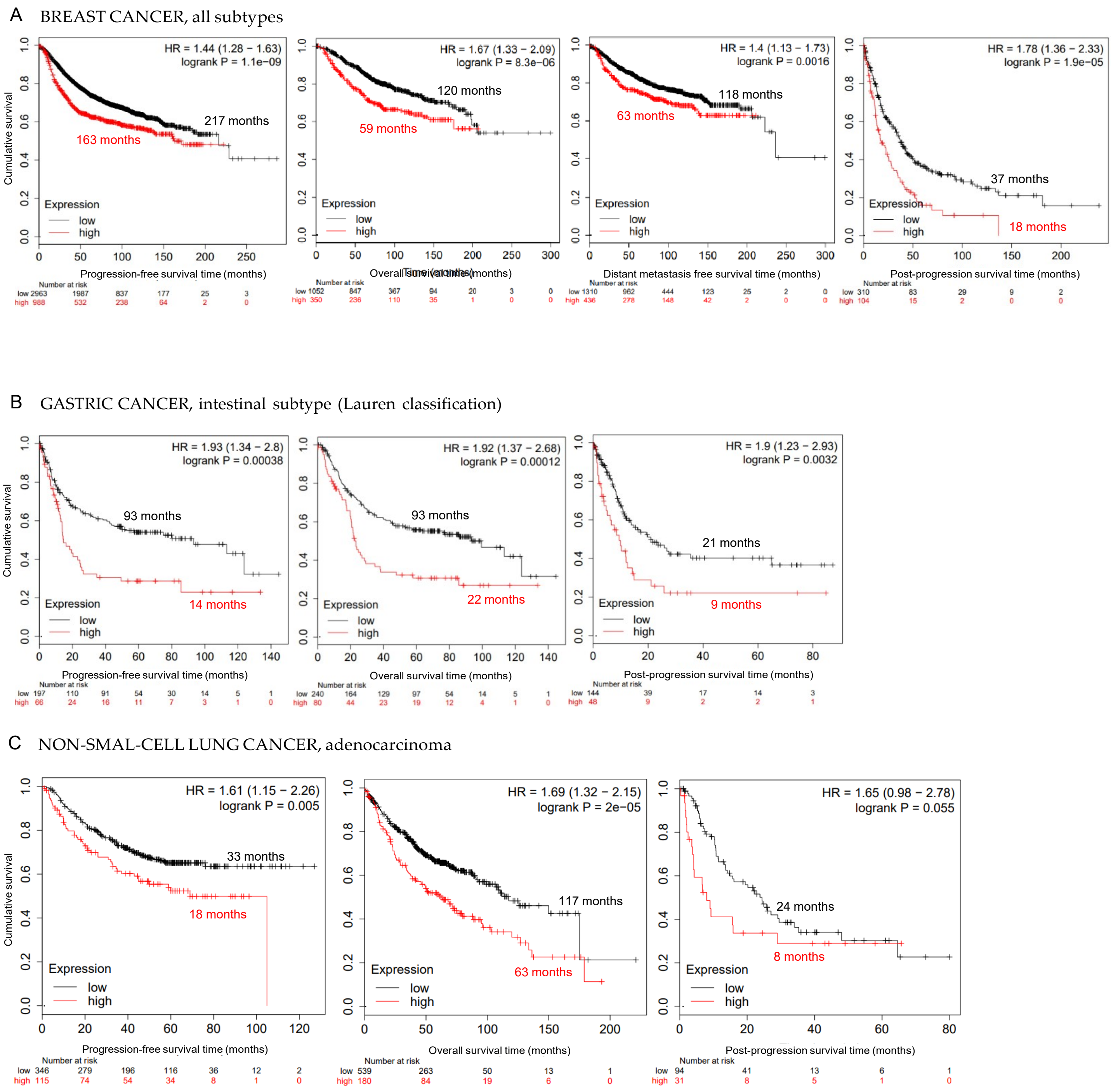

Figure S7: *KRT7* gene expression is associated with poor prognosis in breast, gastric and lung cancer. **A-B**. Kaplan- Meier curves of *KRT7* gene expression associations with reported clinical parameters in breast cancer (all subtypes combined) (**A**), gastric cancer (intestinal subtype according to Lauren classification) (**B**) and non-small-cell lung cancer (adenocarcimoma subtype) (**C**). Gene expression of *KRT7* was dichotomized by the 75th percentile into groups of low and high expression. Log rank p values, hazard ratio (HR) and confidence intervals are indicated. Number of patients and estimated median survival months are reported for each group.

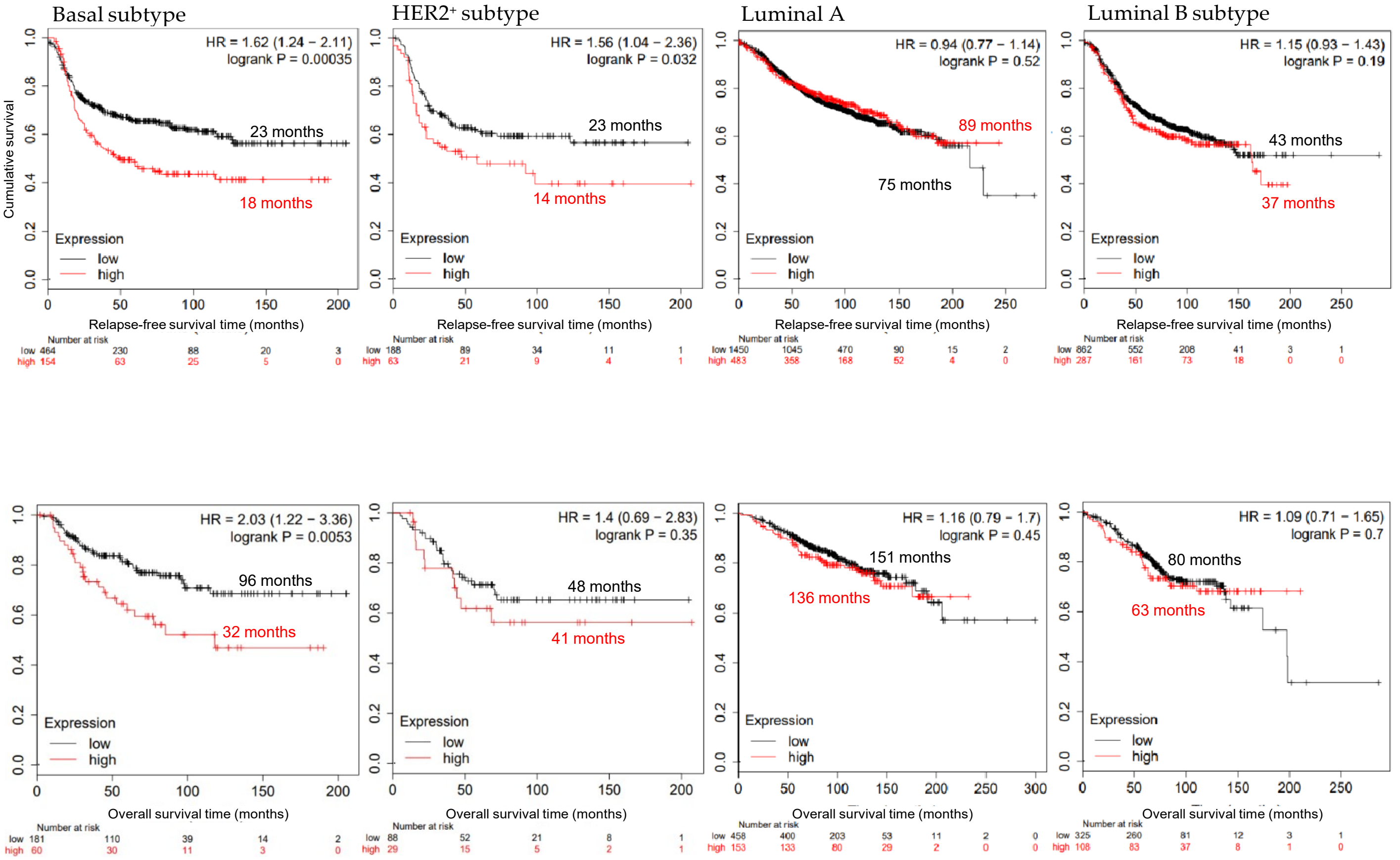

Figure S8: KRT7 high expression is associated with poorer prognosis in the most aggressive breast cancer subtypes. Kaplan-Meier curves of *KRT7* gene expression associations with reported clinical parameters in Kaplan-Meier plotter breast cancer dataset. *KRT7* association with relapse-free survival (up) and overall survival (below) in basal, HER2+, luminal A and luminal B subtype groups of patients. Gene expression of *KRT7* was dichotomized by the 75th percentile into groups of low and high expression. Log rank p values, hazard ratio (HR) and confidence intervals are indicated. Number of patients and estimated median number of survival months are reported for each group.
